# The Human Milk-derived Peptide Drives Rapid Regulation of Macrophage Inflammation Responses in the Neonatal Intestine

**DOI:** 10.1016/j.jcmgh.2024.101420

**Published:** 2024-10-15

**Authors:** Fuqiang Yuan, Xu Han, Masha Huang, Yinglin Su, Yiting Zhang, Mengyuan Hu, Xiang Yu, Weilai Jin, Yun Li, Le Zhang

**Affiliations:** 1 Department of Neonatology, Affiliated Children’s Hospital of Jiangnan University (Wuxi Children’s Hospital), Wuxi, China; 2 Department of Pediatric Laboratory, Affiliated Children’s Hospital of Jiangnan University (Wuxi Children’s Hospital), Wuxi, China; 3 Department of Biochemistry and Molecular Cell Biology, Shanghai Key Laboratory for Tumor Microenvironment and Inflammation, Shanghai Jiao Tong University School of Medicine, Shanghai, China; 4 Department of Neonatology, Affiliated Wuxi Children’s Hospital of Nanjing Medical University, Wuxi, China

**Keywords:** Extracellular Vesicles, FHL2/TRAF6, Human Milk, Macrophage, Peptides

## Abstract

**Background & Aims:**

The interactions between human milk and the regulation of innate immune homeostasis in newborns, and their impact on intestinal health, are not fully understood. This study aimed to explore the role of peptides in human milk extracellular vesicles (EVs) in this process.

**Methods:**

A comprehensive screening of peptides within human milk EVs was performed, leading to the identification of a beta-casein-derived peptide (CASB_135-150_). The effects of CASB_135-150_ on intestinal injury were evaluated in a rat necrotizing enterocolitis (NEC) model. Immunofluorescence analysis was used to determine its distribution, and its impact on NF-κB signaling and inflammation was studied in bone marrow-derived macrophages (BMDMs) and intestinal macrophages. Protein-protein interaction (PPI) analysis, single-cell RNA-seq (scRNA-seq), and co-immunoprecipitation (co-IP) experiments were conducted to explore the mechanism underlying CASB_135-150_ function.

**Results:**

CASB_135-150_ significantly mitigated intestinal injury in the rat NEC model. Immunofluorescence analysis revealed that CASB_135-150_ could target intestinal macrophages and rapidly inhibited NF-κB signaling and reduced inflammation. ScRNA-seq analyses indicated a strong association between FHL2 and NEC development, and co-IP confirmed the interaction between CASB_135-150_ and FHL2. CASB_135-150_ disrupted the FHL2/TRAF6 complex, reducing TRAF6 protein levels. Mutation of key amino acids in CASB_135-150_ disrupted its interaction with FHL2 and abolished its ability to inhibit NF-κB signaling, which also prevented its protective effect in vivo. RNA-seq of intestinal tissue further highlighted the impact of CASB_135-150_ on the NF-κB signaling pathway.

**Conclusions:**

Our study identifies CASB_135-150_, a novel peptide in human milk EVs, that rapidly regulates macrophage inflammatory responses and protects against NEC-induced intestinal injury. These findings provide new insights into the role of human milk in modulating the infant immune system and intestinal health.

## Graphical abstract

**Figure:**
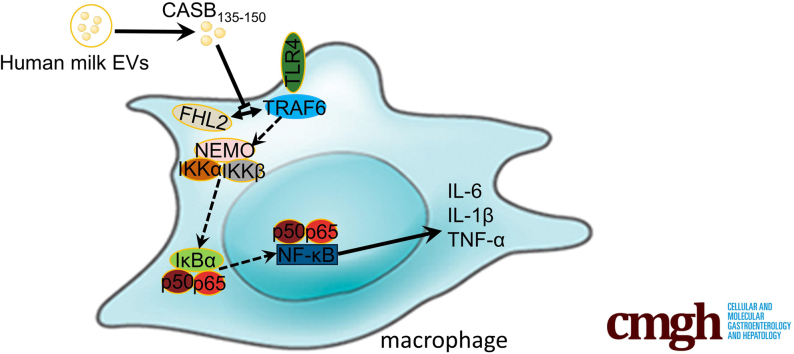

**Summary**

The interactions between human milk and the regulation of innate immune homeostasis in the newborn intestine are not fully understood. Here, we identified a novel human milk-derived peptide, CASB_135-150_, which rapidly modulates macrophage inflammatory responses and protects against necrotizing enterocolitis-induced intestinal injury.

Necrotizing enterocolitis (NEC) continues to pose a significant concern for premature neonates, affecting around 5% to 10% of infants born with a birth weight below 1500 grams.^1^ Particularly in its surgical form, NEC remains a leading cause of morbidity and mortality among premature neonates.^2^

The provision of a premature infant with their mother’s own breast milk is the most potent protective measure against the onset of NEC.^3^^,^^4^ NEC frequently arises from a compromised intestinal barrier, an underdeveloped immune system, and an imbalanced gut microbiome.^5^, ^6^, ^7^ Human milk is recognized as a valuable source of distinctive and dynamic bioactive constituents that play a crucial role in immune system development.^8^ The immune components of human milk include soluble immunoglobulin A (IgA), osteopontin human milk oligosaccharides (HMOs), and activated leukocytes, including neutrophils, macrophages, and T cells.^9^, ^10^, ^11^, ^12^, ^13^ Although the benefits of human milk for nourishing infants are well-established, many questions persist regarding its immune components and their impacts on recipient infants. It is essential to gain an in-depth understanding of the fate and function of these immune agents in newborns and their short-term and long-term effects on immunity.

Human milk is rich in a plethora of bioactive proteins, each serving diverse functions. Recent research has unveiled that some of these activities are not solely ascribed to the parent proteins but are largely attributable to bioactive peptides released from them. Bioactive peptides derived from key human milk proteins, including caseins, α-lactalbumin, and lactoferrin, have been proposed to possess immunomodulatory properties.^14^^,^^15^ Some bioactive peptides exert their effects directly in the gastrointestinal lumen. In addition to proton-dependent peptide transporter (such as PepT1)-mediated intestinal absorption of peptides, a vesicle-mediated transport system is also present.^16^ Notably, after breastfeeding, bioactive peptides derived from human milk proteins are effectively protected against proteases and hydrolysis within the gastrointestinal tract for a specific period, thereby enhancing their efficacy in this particular context.^17^ However, the functional mechanisms of human milk-derived vesicle-mediated peptides in gastrointestinal immune regulation remain largely uncharted.

In this study, we identified a peptide derived from milk vesicles that effectively and rapidly suppresses pro-inflammatory responses in macrophages during NEC development. Additionally, our investigation reveals that this peptide interacts with FHL2 and disrupts the FHL2/TRAF6 complex, consequently inhibiting NF-κB signaling activity upon exposure to lipopolysaccharide (LPS). These findings shed light on a previously unknown endogenous regulator of NF-κB signaling and provide valuable insights into the intricate relationship between maternal feeding and the regulation of the infant’s intestinal innate immune system.

## Results

### β-casein-derived Peptide CASB_135-150_ Demonstrates a Protective Effect Against NEC

**Figure 1:**
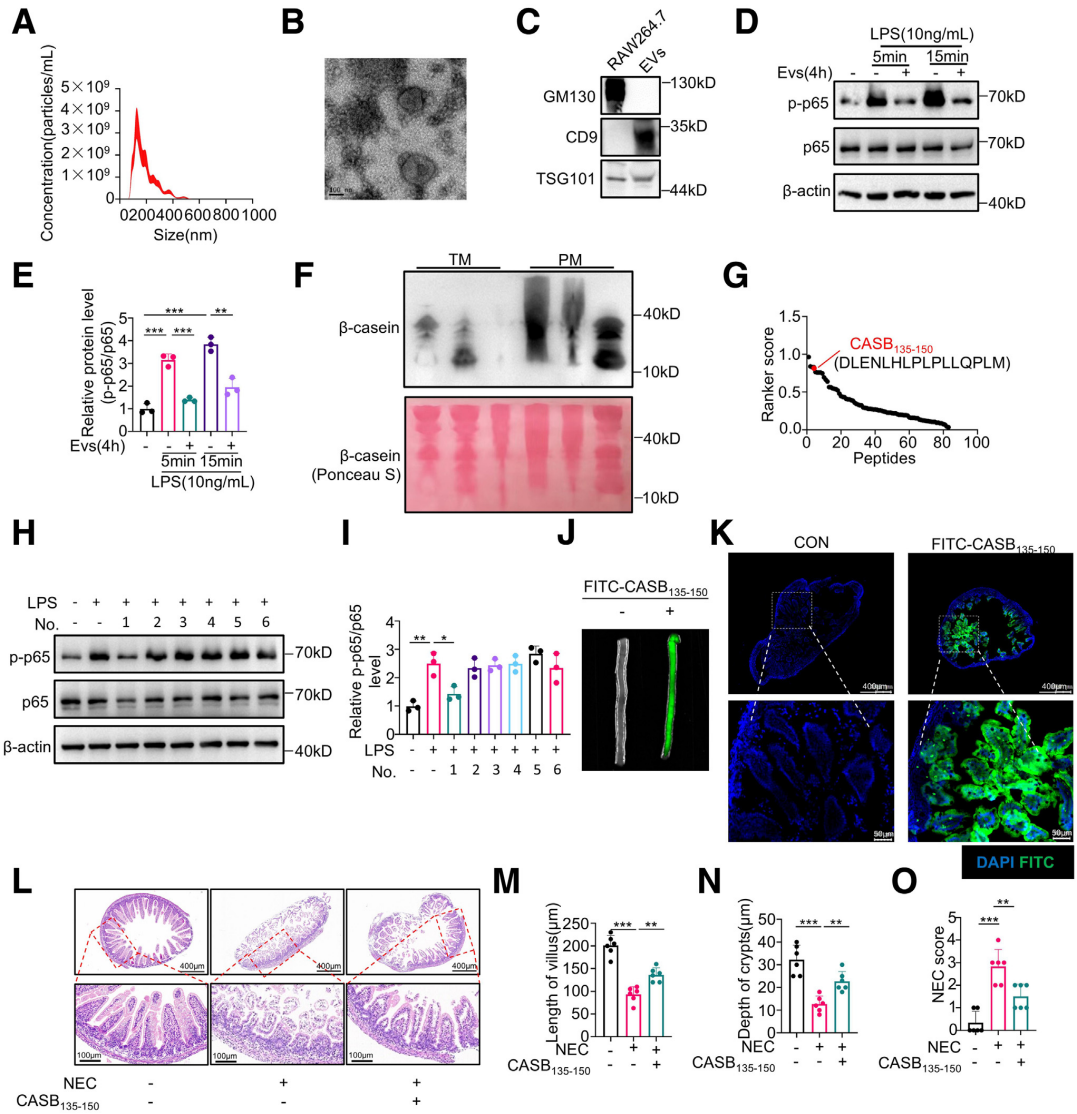
**β-casein-derived peptide CASB**_**135-150**_**demonstrates a protective effect against NEC.** (*A–C*) Milk EVs were isolated from human colostrum and characterized using a combination of NTA (*A*), TEM (*B*), and Western blotting against CD9, TSG101, and GM130 proteins (*C*). (*D–E*) BMDMs were pre-treated with milk EVs for 4 hours, followed by stimulation with LPS (10 ng/mL) for varying time intervals. Western blotting was performed to examine the p-p65 and p65 (*D*), and the quantification results are shown in (*E*), n = 3. (*F*) Milk EVs proteins were extracted for Tricine-SDS-PAGE analysis against β-casein. “TM” stands for colostrum derived from the mother of a term infant, and “PM” stands for colostrum derived from the mother of a preterm infant. (*G*) The peptide bioactivity within milk EVs were assessed in silico using the PeptideRanker online server. (*H–I*) BMDMs were pretreated with the indicated peptides (50 μM) for 1 hour, followed by stimulation with LPS (10 ng/mL) for 5 minutes. Subsequently, Western blotting was performed to analyze the activation of p65 (*H*), and the quantification results are shown in (*I*), n = 3. (*J–K*) The neonatal rats received intraperitoneal injections of FITC-CASB_135-150_ (20 mg/kg). After 8 hours, the ileum tissue (*J*) and ileum sections (*K*) were subjected for fluorescence imaging. (*L-O*) The neonatal rats received intraperitoneal injections of CASB_135-150_ (20 mg/kg) once a day, whereas the NEC model was being established. n = 6. The small intestines were harvested for the determinations of H&E staining (*L*), villus length (*M*), crypts depth (*N*), and NEC score (*O*). Data are presented as mean ± SD. Statistical significance was determined using 1-way ANOVA with Tukey’s post hoc test. ∗*P* < .05; ∗∗*P* < .01; ∗∗∗*P* < .001.

Milk extracellular vesicles (EVs) were isolated from human breast milk and characterized using a combination of nanoparticle tracking analysis (NTA), transmission electron microscopy (TEM), and Western blotting against TSG101, CD9, and GM130 proteins (Figure 1*A–C*). The milk EVs primarily exhibit a size distribution ranging from 100 to 400 nm, meeting the specified criteria delineating microvesicles (100 to 1000 nm).^18^^,^^19^ To explore the impact of breast milk EVs on the immune response of macrophages, bone marrow-derived macrophages (BMDMs) were pre-treated with these EVs, followed by stimulation with LPS for varying time intervals. Notably, a 4-hour pre-exposure to EVs exhibited a noteworthy inhibition of NF-κB p65 Ser536 phosphorylation (p-p65) (Figure 1*D–E*). This phenomenon underscores the potential of breast milk-derived EVs to modulate macrophage immune response.

Breast milk EVs, known to harbor diverse peptides, could potentially serve as rapid response effectors in the modulation of both physiological and pathological signaling pathways.^20^ In a prior investigation, a thorough peptidome analysis of breast milk EVs was conducted and identified several peptides derived from β-casein.^20^ The utilization of Tricine- sodium dodecyl sulfate-polyacrylamide gel electrophoresis (SDS-PAGE) analysis effectively unveiled β-casein-derived peptides with varying molecular weights (Figure 1*F*). Strikingly, an augmented abundance of these peptides was discernible in EVs of preterm milk when contrasted with their levels in term milk EVs (Figure 1*F*). Among the β-casein-derived peptides, 6 peptide sequences exhibited a considerable likelihood of bioactivity, as predicted through the application of Peptide Ranker (Figure 1*G*; Table 1).

**Table 1:** The Peptide Sequences Derived From β-casein

Number	Peptide sequence	Ranker score
1	DLENLHLPLPLLQPLM	0.805159
2	YPFVEPIPYGFLP	0.829812
3	KSPTIPFFD	0.765449
4	YPFVEPIPYGFLPQN	0.69394
5	IYPSFQPQPLIYPFVEPI	0.566071
6	QQVPQPIPQTL	0.564323

Subsequently, these peptides were synthesized with an N-terminal attachment of the cell-penetrating peptide TAT to facilitate functional screening, with p-p65 serving as the readout.^21^ Although treatment with other peptides showed negligible impact on p-p65, pretreatment with the No. 1 peptide markedly suppressed this signal (Figure 1*H–I*). To facilitate future research, Peptide No. 1 has been renamed to CASB_135-150_, which is derived from the 135-150 amino acid sequence of beta-casein. To further confirm its function *in vivo*, FITC-labeled CASB_135-150_ was synthesized and intraperitoneally into neonatal rats to trace its distribution. Fluorescence signals were detected in the ileum 8 hours after injection (Figure 1*J*). Additionally, pathological sections of the ileum demonstrated that the peptides could be detected in the intestinal villus (Figure 1*K*), confirming effective penetration into NEC lesions.

**Figure 2:**
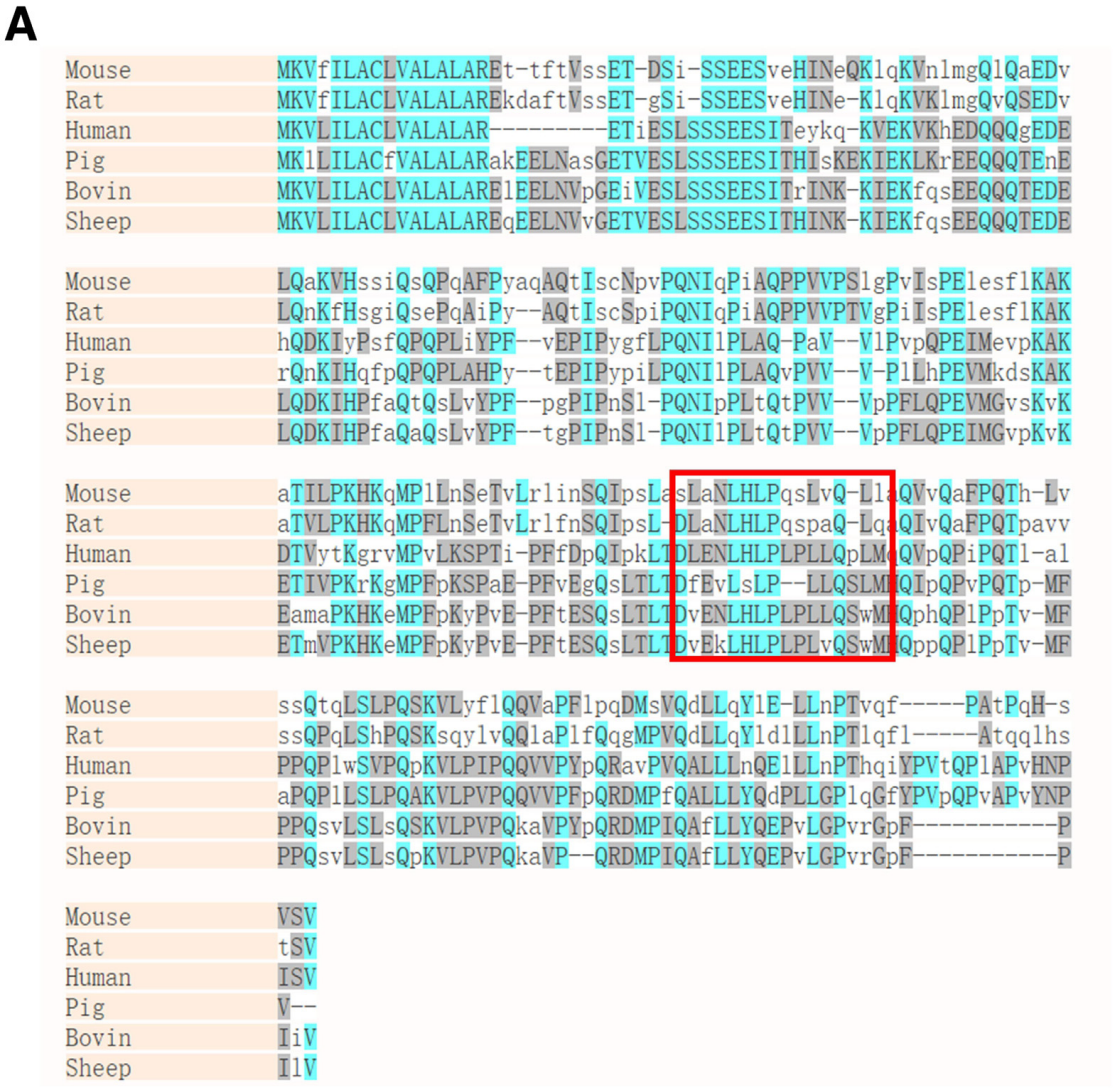
**CASB**_**135-150**_**exhibits a relatively high degree of conservation among mammals.** (*A*) The amino acid sequence alignment among mammals.

Notably, the consistent trend was upheld in an *in vivo* study utilizing a rat model of NEC, wherein administration of the CASB_135-150_ ameliorated intestinal villous structural damage and restored villus length and crypts depth (Figure 1*L–O*). Importantly, the CASB_135-150_ exhibits a relatively high degree of conservation among mammals (Figure 2). Taken together, these findings underscore the abundance of β-casein-derived peptides in breast milk EVs and reveal that CASB_135-150_ may play a significant role in regulating inflammatory responses in macrophages.

### CASB_135-150_ Mitigates the Pro-inflammatory Response in Intestinal Macrophages

**Figure 3:**
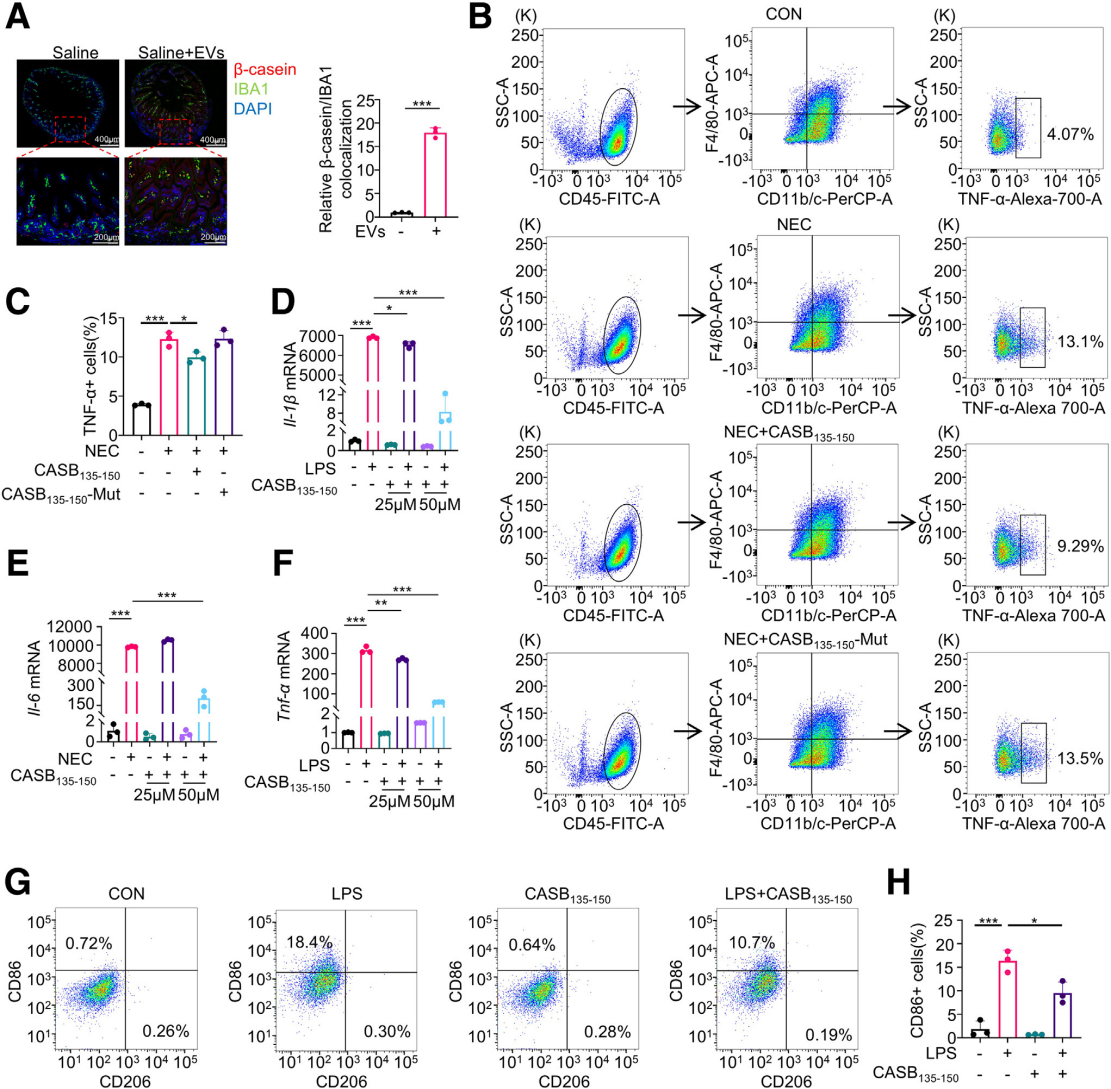
**CASB**_**135-150**_**mitigates the pro-inflammatory response in intestinal macrophages.** (*A*) The neonatal rats were gavaged with either saline or saline supplementation with human milk EVs. After a 2-hour period, the ileums were collected for immunofluorescence analysis of IBA1, β-casein, and DAPI. n = 3. (*B–C*) Intestinal lamina propria lymphocytes were isolated from NEC animals that received CASB_135-150_ or CASB_135-150_-Mut treatment (20 mg/kg/day for 4 days). CD45+F4/80+CD11b/c+ macrophages were gated for analysis of TNF-α by flow cytometry (*B*) and the percentage of TNF-α+ cells quantification, n = 3 (*C*). (*D–F*) BMDMs were pre-treated with CASB_135-150_ at the indicated concentrations for 1 hour, followed by stimulation with LPS (10 ng/ml) for 6 hours. Subsequently, the levels of inflammation factors were assessed using qRT-PCR, GAPDH as a reference gene, n = 3. (*G–H*) BMDMs were pre-treated with CASB_135-150_ at a concentration of 50 μM, followed by LPS (10 ng/ml) treatment for 24 hours. Afterward, the cells were collected for flow cytometry analysis of CD86 and CD206 (*G*) and quantification (*H*). n = 3 Data are presented as mean ± SD. Statistical significance was determined using 1-way ANOVA with Tukey’s post hoc test. ∗*P* < .05; ∗∗*P* < .01; ∗∗∗*P* < .001.

**Figure 4:**
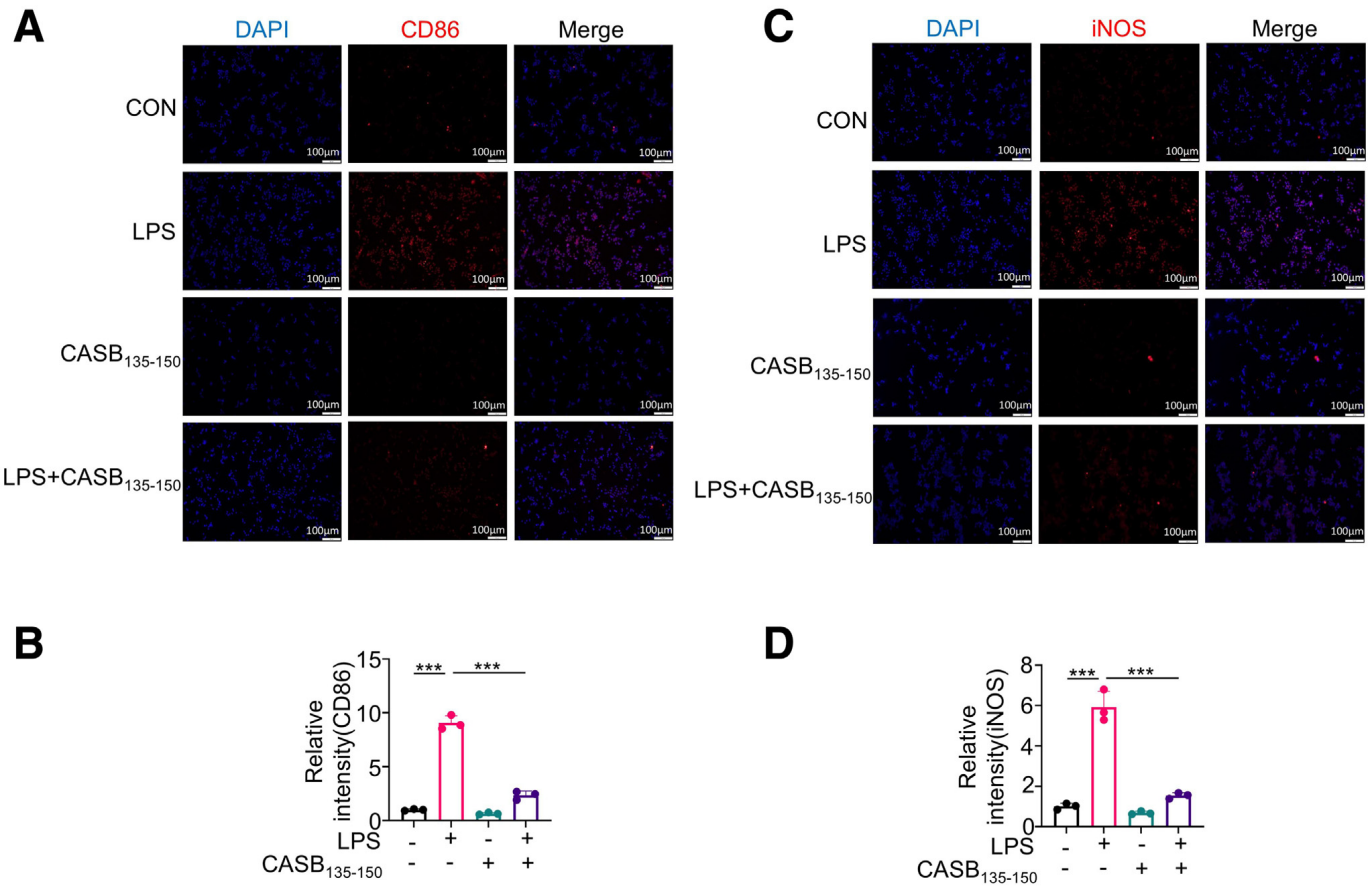
**CASB**_**135-150**_**inhibits the pro-inflammation phenotype of macrophage.** (*A–D*) RAW264.7 cells were pre-treated with CASB_135-150_ (50 μM) for 1 hour, followed by exposure to LPS (10 ng/ml) for 12 hours. Subsequently, immunofluorescence was conducted to assess CD86 and iNOS. n = 3. Data are presented as mean ± SD. Statistical significance was determined using 1-way ANOVA with Tukey’s post hoc test. ∗*P* < .05; ∗∗*P* < .01; ∗∗∗*P* < .001.

To investigate the *in vivo* distribution of β-casein-related peptides encapsulated within EVs, neonatal rats were administered saline supplemented with milk EVs via gavage. Notably, after oral administration of the EVs, there is an obvious colocalization of beta-casein and macrophages (Figure 3*A*). Particularly, the administration of EVs results in an augmented fluorescence signal intensity of β-casein within macrophages (Figure 3*A*). To explore the function of CASB_135-150_ in intestinal macrophages, intestinal lamina propria lymphocytes were isolated, and CD45+F4/80+CD11b/c+ macrophages were gated for analysis of tumor necrosis factor alpha (TNF-α) by flow cytometry (Figure 3*B*). There is a significant increase in the TNF-α portion in the NEC group compared with the control group. Additionally, CASB_135-150_ effectively reduced the percentage of TNF-α+ macrophages, whereas CASB_135-150_-Mut (this will be mentioned below) had no significant effect (Figure 3*B–C*). Consistently, CASB_135-150_ treatment reduced the levels of CD86 and iNOS that had been increased by LPS in BMDMs (Figure 4).

**Figure 5:**
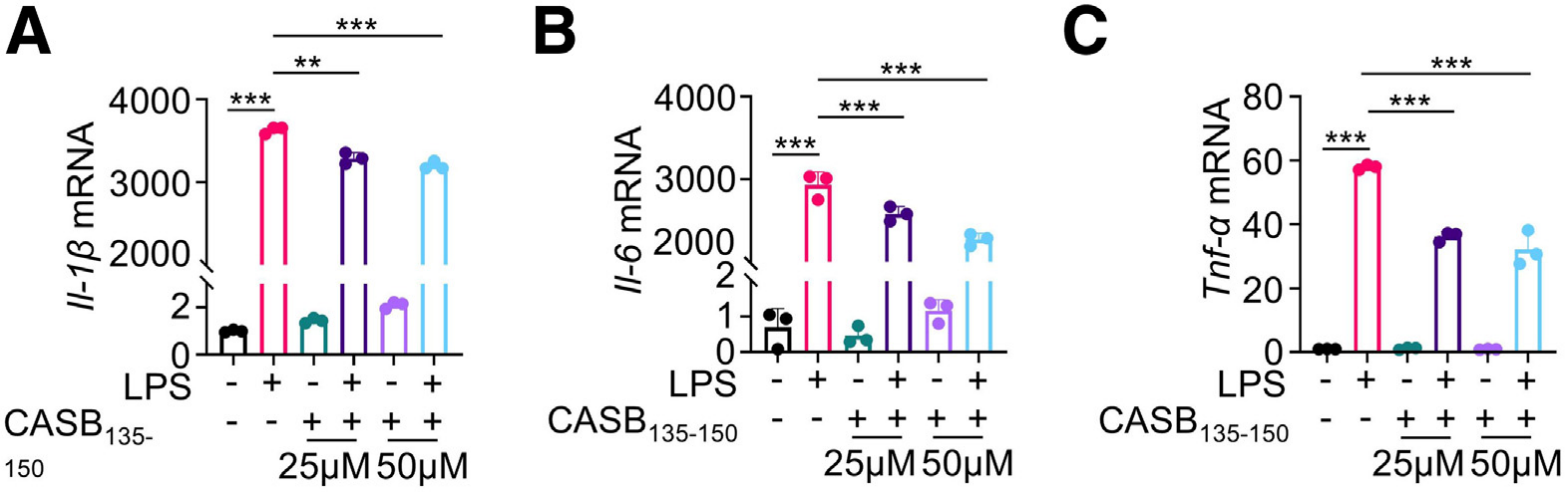
**CASB**_**135-150**_**suppresses the inflammatory response of macrophages in response to LPS.** (*A–C*) RAW264.7 cells were pre-treated with CASB_135-150_ at the indicated concentrations for 1 hour, followed by stimulation with LPS (10 ng/mL) for 6 hours. Subsequently, the levels of inflammation factors were assessed using qPCR, n = 3. Data are presented as mean ± SD. Statistical significance was determined using 1-way ANOVA with Tukey’s post hoc test. GAPDH as a reference gene. ∗*P* < .05; ∗∗*P* < .01; ∗∗∗*P* < .001.

The application of CASB_135-150_ resulted in a significant decrease in the production of pro-inflammatory cytokines, including *Il-1β*, *Il-6*, and *Tnf-α*, induced by LPS in BMDMs and RAW264.7 cells (Figure 3*D–F*; Figure 5*A–C*). Furthermore, employing flow cytometry, the expression levels of CD86 and CD206, markers indicative of M1 and M2 macrophage polarization, were assessed. As expected, treatment with CASB_135-150_ led to a reduction in the proportion of CD86-positive cells, declining from approximately 18% to around 10% when compared with cells exposed solely to LPS (Figure 3*G–H*). Nevertheless, it is worth noting that CASB_135-150_ treatment did not exhibit any discernible impact on the proportion of M2 macrophages (Figure 3*G–H*). Collectively, these findings collectively propose that CASB_135-150_ effectively suppresses the inflammatory response of macrophages, which could potentially contribute to protecting against neonatal colitis.

### CASB_135-150_ Promotes the Proliferation of Intestinal Cells

**Figure 6:**
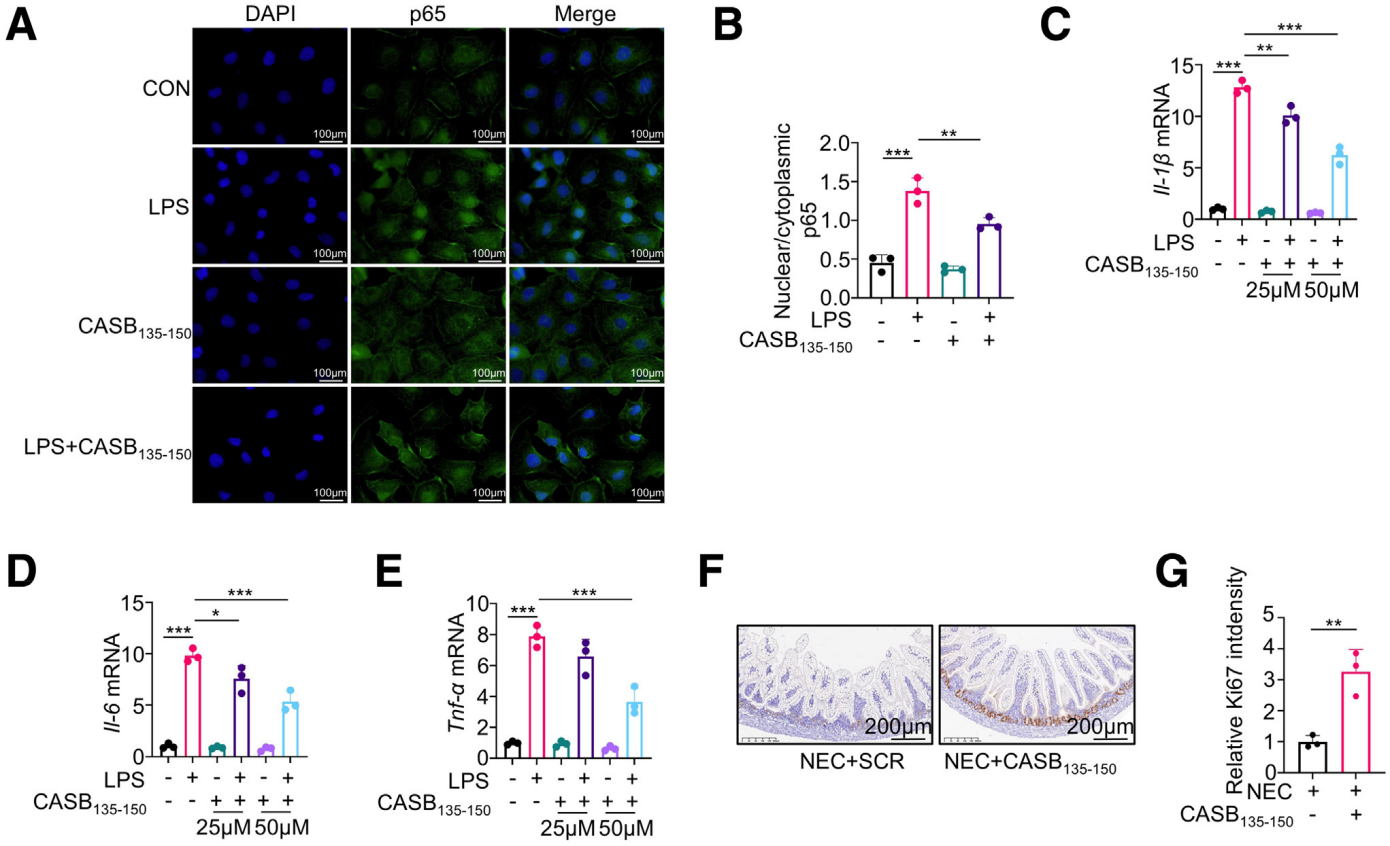
**CASB**_**135-150**_**promotes the proliferation of intestinal cells.** (*A–B*) IEC6 cells were pre-treated with CASB_135-150_ (50 μM) for 1 hour, followed by exposure to LPS (10 ng/ml) for 2 hours. Subsequently, immunofluorescence was conducted to assess the translocation of p65 (*A*). The quantification was carried out by calculating the nuclear p65 to cytoplasmic p65 ratio (*B*). n = 3. (*C–E*) IEC6 cells were pre-treated with CASB_135-150_ at the indicated concentrations for 1 hour, followed by stimulation with LPS (10 ng/ml) for 6 hours. Subsequently, the levels of inflammation factors were assessed using qRT-PCR, GAPDH as a reference gene, n = 3. (*F–G*) Ki67 staining was used to identify newly proliferative cells in the ileum segments of NEC rats following intervention with CASB_135-150_ or scrambled peptide (*F*), and the quantification of Ki67-positive area was performed (*G*). Statistical significance was determined using 1-way ANOVA with Tukey’s post hoc test. GAPDH as a reference gene. ∗*P* < .05; ∗∗*P* < .01; ∗∗∗*P* < .001.

To gain a more comprehensive understanding of CASB_135-150_’s role in NEC, we assessed its function in rat intestinal mucosal IEC-6 cell line. IEC-6 cells were treated with LPS ± CASB_135-150_, and p65 translocation was examined. Consistent with observations in macrophages, CASB_135-150_ effectively reduced p65 nuclear translocation following LPS treatment (Figure 6*A–B*). Additionally, the increased expression of Il-6, Il-1β, and Tnf-α induced by LPS was significantly reduced by CASB_135-150_ treatment (Figure 6*C–E*). As FHL2 is also expressed in epithelial cells and involved in the inflammatory response, CASB_135-150_ may exert its function in these cells as well. We also examined Ki67 staining in ileum sections. It was shown that CASB_135-150_ could increase the presence of Ki67-positive proliferative cells in the intestine (Figure 6*F–G*).

### CASB_135-150_ Rapidly Suppresses NF-κB Signaling Activity by Interacting With FHL2

**Figure 7:**
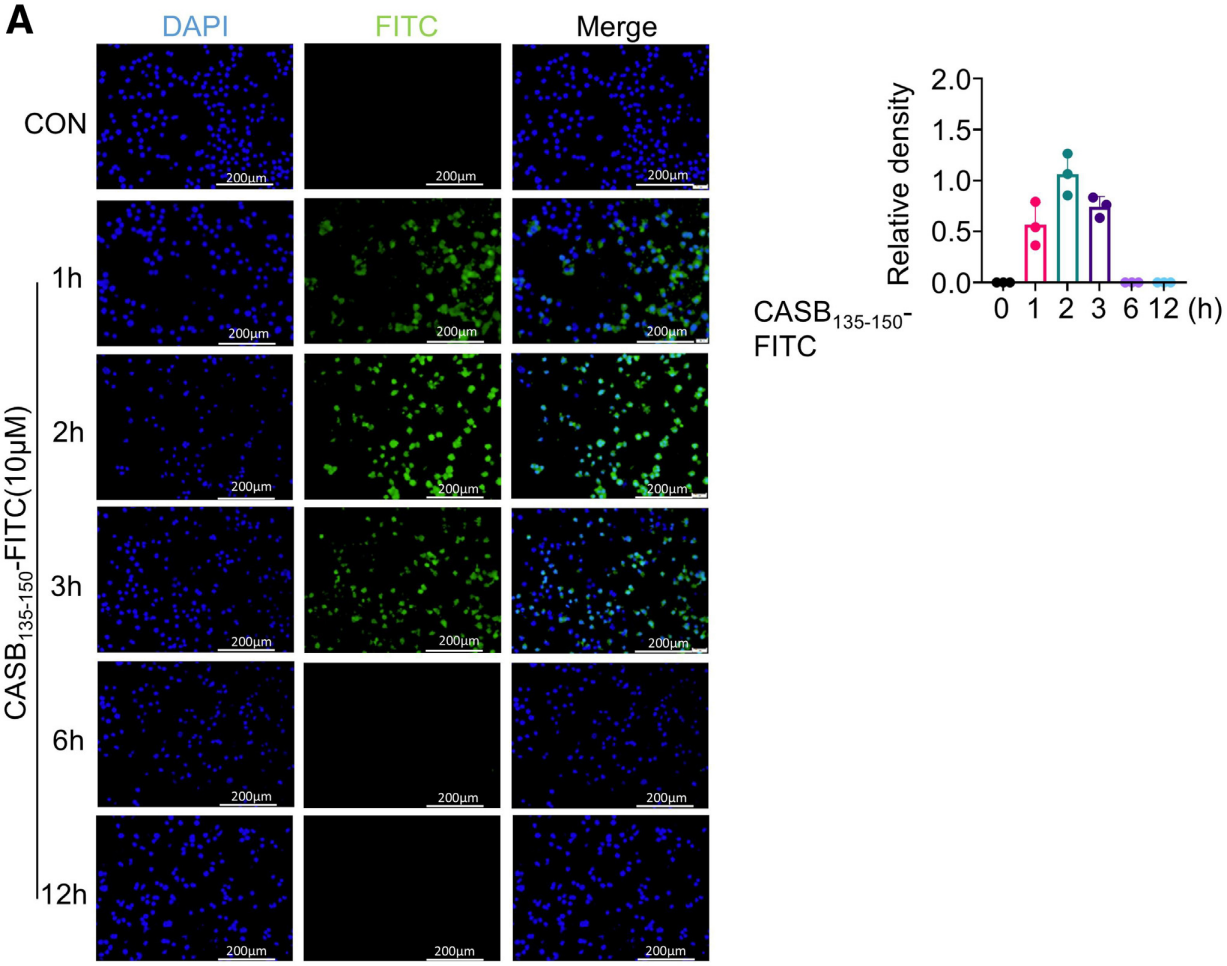
**CASB**_**135-150**_**penetrated into cells effectively.** CASB_135-150_ was synthesized with an N-terminal attachment of a FITC label, termed as CASB_135-150_-FITC. (*A*) This peptide was introduced into the culture of BMDMs, and fluorescence was monitored at various time points.

**Figure 8:**
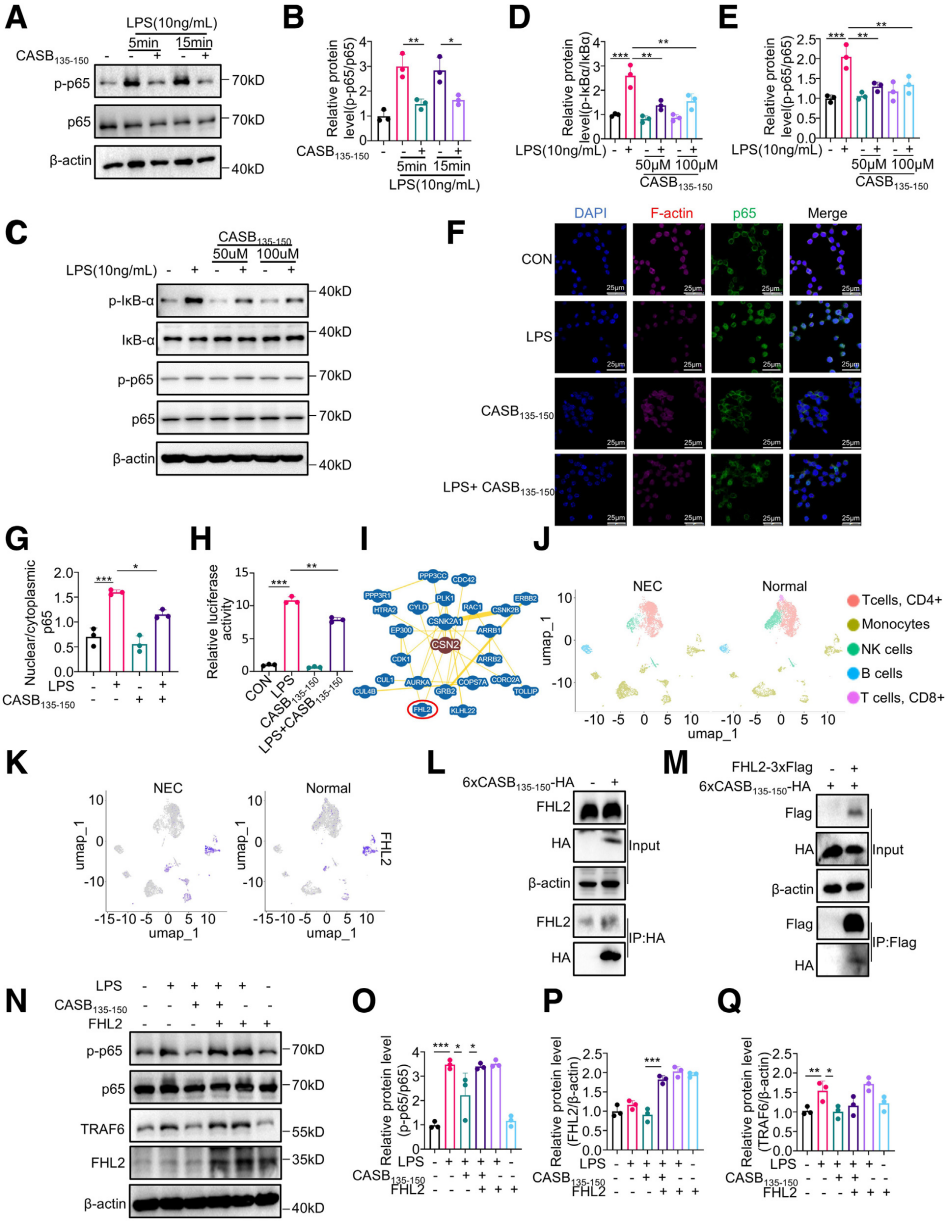
**CASB**_**135-150**_**rapidly suppresses NF-κB signaling activity by interacting with FHL2.** (*A–B*) BMDMs were pre-treated with CASB_135-150_ (50 μM) for 1 hour, followed by acute exposure to LPS (10 ng/ml) for the specified duration. Subsequently, the cells were harvested for Western blotting analysis of p-p65 (*A*) and quantification (*B*). n = 3. (*C–E*) BMDMs were pre-treated with CASB_135-150_ at various concentrations, followed by treatment with LPS (10 ng/ml) for 5 minutes. Total protein was then extracted for western blotting analysis (*C*) and quantifications (*D, E*). n = 3. (*F–G*) RAW264.7 cells were pre-treated with CASB_135-150_ (50 μM) for 1 hour, followed by exposure to LPS (10 ng/ml) for 2 hours. Subsequently, immunofluorescence was conducted to assess the translocation of p65 (*F*). The quantification was carried out by calculating the nuclear p65 to cytoplasmic p65 ratio (*G*). n = 3. (*H*) RAW264.7 cells were co-transfected with the NF-κB luciferase reporter construct and the Renilla luciferase construct. The cells were then pre-treated with CASB_135-150_ (50 μM) for 1 hour, followed by LPS (10 ng/ml) treatment for 3 hours. Subsequently, the cells were harvested for dual-luciferase analysis. n = 3. (*I*) The β-casein/CSN2 interactome was established using the STRING online website. (*J–K*) The single-cell transcriptomic analysis was conducted using a previously published dataset that encompasses NEC and normal human neonatal small intestine tissues, revealing distinct clusters of immune cells (*J*). The expression distribution of FHL2 across each cell cluster (*K*). (*L*) RAW264.7 cells were overexpressed with 6×CASB_135-150_-HA, followed by the anti-HA co-IP. (*M*) The FHL2-3×Flag and 6×CASB_135-150_-HA were co-transfected into RAW264.7 cells, and co-IP was carried out using an anti-Flag antibody. (*N–Q*) RAW264.7 cells were overexpressed with FHL2 for 48 hours. Subsequently, they were treated with CASB_135-150_ (50 μM) for 1 hour, followed by LPS (10 ng/ml) treatment for 5 minutes. The cells were then harvested for Western blotting analysis (*N*) and quantifications (*O–Q*). n = 3. Data are presented as mean ± SD. Statistical significance was determined using 1-way ANOVA with Tukey’s post hoc test. ∗*P* < .05; ∗∗*P* < .01; ∗∗∗*P* < .001.

To investigate the impact of CASB_135-150_ on the inflammatory response of macrophages, BMDMs were pre-treated with CASB_135-150_ for a duration of 1 hour (as 1 hour is the minimum time for peptide entry into the cells, as shown in Figure 7), followed by acute exposure to LPS. Notably, the swift activation of p65 induced by LPS was strongly suppressed following CASB_135-150_ preconditioning (Figure 8*A–B*). Employing a concentration gradient of CASB_135-150_ for treatment, it was observed that 50 μM CASB_135-150_ has achieved the maximum inhibitory effect on NF-κB (Figure 8*C–E*). Moreover, the translocation of p65 to the nucleus triggered by LPS was also repressed by CASB_135-150_ treatment (Figure 8*F–G*). Through employment of a dual-luciferase reporter assay system, CASB_135-150_ demonstrated a significant reduction in NF-kB promoter luciferase activity (Figure 8*H*). Collectively, these observations suggest that CASB_135-150_ rapidly inhibits NF-κB signaling, likely functioning predominantly upstream of p65.

**Figure 9:**
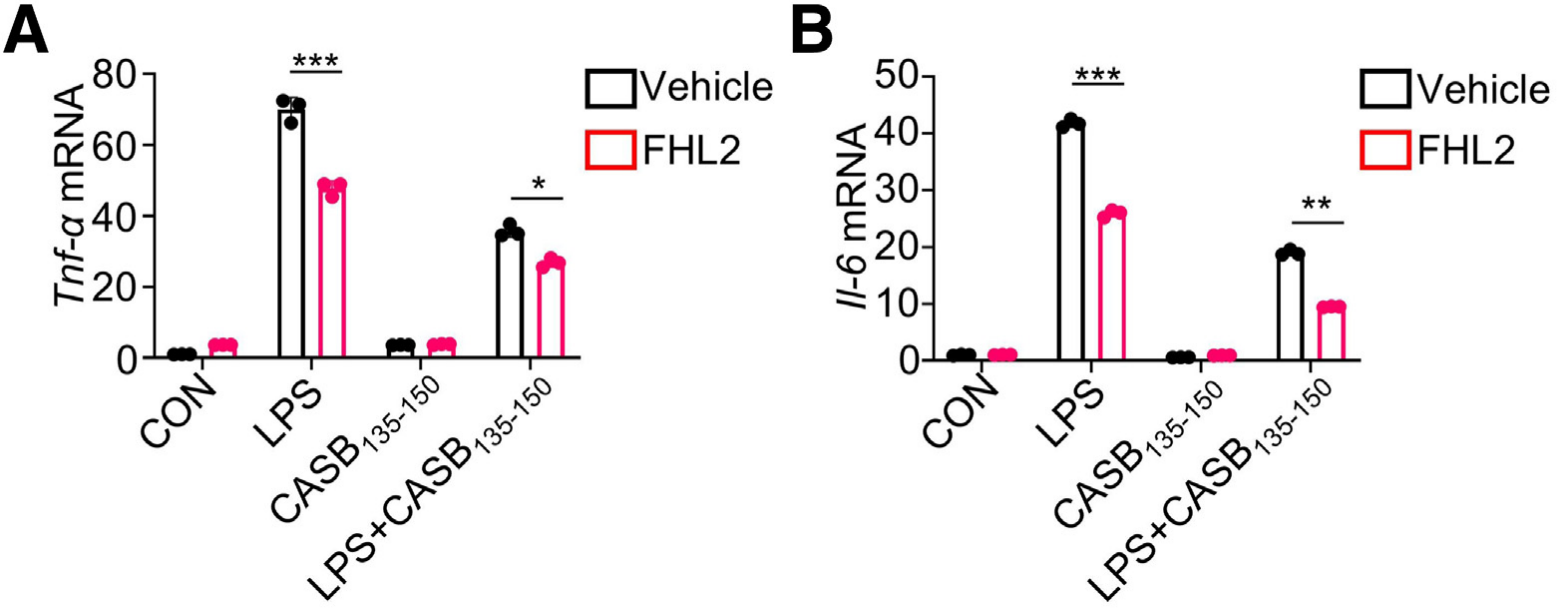
**FHL2 overexpression amplifies the anti-inflammatory effects controlled by CASB**_**135-150**_**treatment.** (*A–B*) RAW264.7 cells were overexpressed with FHL2 for 48 hours, followed by pre-treatment with CASB_135-150_ for 1 hour and subsequent exposure to LPS for 6 hours. The production of Tnf-α (*A*) and IL-6 (*B*) was assessed using qPCR. GAPDH as a reference gene. ∗*P* < .05; ∗∗*P* < .01; ∗∗∗*P* < .001.

Given that endogenous peptides often retain certain structural and functional characteristics of their precursor proteins, we proceeded to analyze the interactome of β-casein to investigate the potential functional target of CASB_135-150_ (Figure 8*I*). Among the interactive proteins, FHL2 is a regulating factor upstream of NF-κB signal.^22^^,^^23^ In addition, based on a single-celled omics analysis,^24^ FHL2 was increased in the population of monocytes in NEC intestine (Figure 8*J–K*). Indeed, through overexpressing 6 copies of CASB_135-150_ with a HA tag (6 × CASB_135-150_-HA) (Table 2), followed by the anti-HA co-immunoprecipitation (co-IP), the result showed that CASB_135-150_ is able to interact with FHL2 (Figure 8*L*). Consistently, co-overexpression of FHL2-Flag and 6 × CASB_135-150_-HA, and co-IP using Flag antibody showed that FHL2 could also pull down CASB_135-150_ (Figure 8*M*). To investigate the role of FHL2 in CASB_135-150_-mediated p65 repression, we introduced ectopic expression of FHL2 into BMDMs. As anticipated, FHL2 overexpression notably reversed the decrease in p-p65 induced by CASB_135-150_ treatment (Figure 8*N–Q*). It is worth noting that FHL2 overexpression alone did not significantly affect the levels of p-p65. Additionally, although CASB_135-150_ reduced the production of inflammatory factors, FHL2 overexpression enhanced this effect (Figure 9*A–B*). In conclusion, these results strongly indicate that CASB_135-150_ interacts with FHL2, leading to the inhibition of NF-κB signal activity.

**Table 2:** The Sequence of 6×CASB_135-150_-HA and 6×CASB_135-150_(Mut)-HA

The sequence of 6×CASB_135-150_-HA: ATGGACCTGGAGAACCTGCACCTGCCCCTGCCCCTGCTGCAGCCTCTGATGGGCAGCGGCTCCGATCTGGAGAACCTCCACCTGCCCCTCCCTCTGCTGCAGCCCCTGATGGGCTCTGGCTCCGACCTGGAGAATCTGCACCTGCCTCTGCCTCTGCTGCAACCTCTGATGGGAAGCGGCTCCGACCTCGAGAACCTGCATCTGCCCCTGCCACTGCTGCAGCCACTGATGGGCTCCGGCAGCGATCTGGAGAATCTCCACCTGCCACTGCCCCTGCTCCAGCCTCTGATGGGGAGCGGCTCCGATCTCGAGAACCTCCATCTGCCCCTCCCACTGCTGCAACCACTGATGGGATCTGGCAGCTACCCCTACGATGTGCCCGACTACGCCTGA
The sequence of 6×CASB_135-150_(Mut)-HA:ATG GATCTGGAGAACGCCGCCGCCGCCCTGCCACTGCTGCAACCTCTGATGGGCAGCGGCTCCGATCTGGAGAATGCCGCCGCCGCTCTGCCTCTGCTGCAGCCTCTGATGGGATCTGGCTCCGACCTGGAGAACGCTGCCGCCGCTCTCCCTCTGCTGCAACCACTGATGGGCTCTGGCTCCGATCTCGAGAACGCCGCTGCCGCCCTGCCTCTGCTCCAGCCTCTGATGGGGTCCGGCTCCGACCTCGAGAACGCTGCTGCCGCCCTCCCTCTGCTCCAACCCCTGATGGGCTCCGGCAGCGACCTGGAGAATGCTGCCGCCGCCCTCcCCCTGCTGCAGCCCCTGATGGGAAGCGGCAGCTACCCCTACGACGTGCCCGACTACGCCTGA

### CASB_135-150_ Deconstructs FHL2/TRAF6 Complex to Suppress Inflammation Response

**Figure 10:**
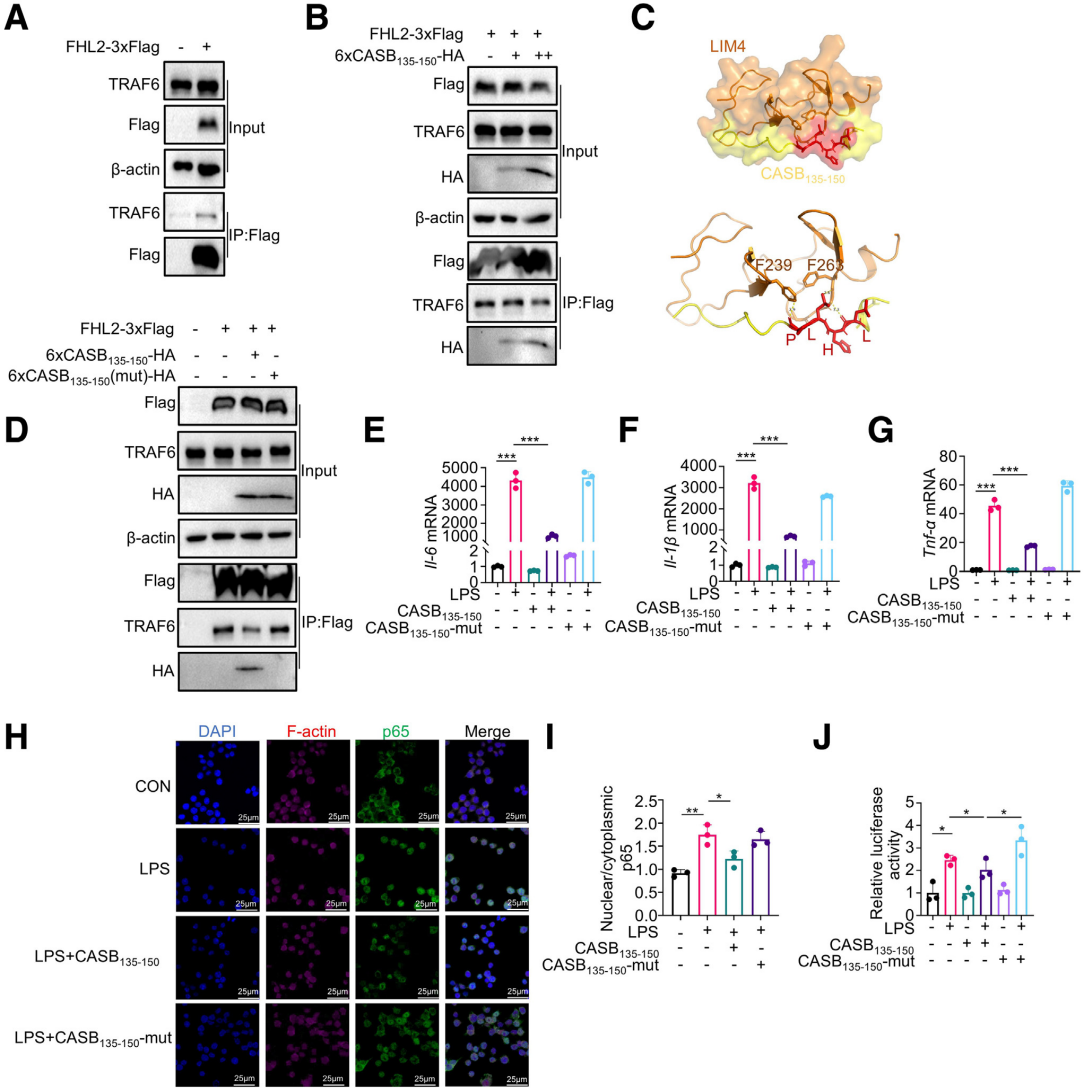
**CASB**_**135-150**_**deconstructs FHL2/TRAF6 complex to suppress inflammation response.** (*A*) RAW264.7 cells were overexpressed with FHL2-3×Flag for 48 hours, and co-IP was carried out using an anti-Flag antibody. (*B*) The FHL2-3×Flag and 6×CASB_135-150_-HA were co-transfected into RAW264.7 cells, and co-IP was carried out using an anti-Flag antibody. (*C*) To simulate the interaction between FHL2 and CASB_135-150_, the molecular docking was performed with LIM4 domain of FHL2 and CASB_135-150_. (*D*) RAW264.7 cells were transfected with constructs as indicated. The co-IP was performed using an anti-Flag antibody. (*E–G*) BMDMs were pre-treated with synthetic CASB_135-150_ (50 μM) or CASB_135-150_-Mut (50 μM) for 1 hour, followed by LPS (10 ng/ml) treatment for 6 hours. Subsequently, the cells were harvested for qPCR analysis of inflammation factors, GAPDH as a reference gene. n = 3. (*H–I*) RAW264.7 cells were pre-treated with CASB_135-150_ (50 μM) or CASB_135-150_-Mut (50 μM) for 1 hour, followed by exposure to LPS (10 ng/ml) for 2 hours. Subsequently, immunofluorescence was conducted to assess the translocation of p65 (*H*). The quantification was carried out by calculating the nuclear p65 to cytoplasmic p65 ratio (*I*). n = 3. (*J*) RAW264.7 cells were co-transfected with the NF-κB luciferase reporter construct and the Renilla luciferase construct. The cells were then pre-treated with CASB_135-150_ (50 μM) or CASB_135-150_-Mut (50 μM) for 1 hour, followed by LPS (10 ng/ml) treatment for 3 hours. Subsequently, the cells were harvested for dual-luciferase analysis. n = 3. Data are presented as mean ± SD. Statistical significance was determined using 1-way ANOVA with Tukey’s post hoc test. ∗*P* < .05; ∗∗*P* < .01; ∗∗∗*P* < .001.

TNF receptor-associated factor 6 (TRAF6) is a known binding partner of FHL2, and they play a pivotal role in the NF-κB pathway.^23^ In line with this, upon the overexpression of FHL2-Flag, co-IP using the Flag antibody revealed the ability of FHL2 to interact with TRAF6 (Figure 10*A*). Moreover, a study by Dahan et al demonstrated that FHL2 stabilizes TRAF6 and enhances its transcriptional activation activity in the NF-κB pathway.^25^ Notably, CASB_135-150_ treatment resulted in a reduction in the abundance of TRAF6 (Figure 8*N*, *Q*). To assess the impact of CASB_135-150_ treatment on the FHL2/TRAF6 complex, FHL2-Flag and CASB_135-150_-HA were co-overexpressed in 293T cells, followed by co-IP using the Flag antibody. Interestingly, as the level of CASB_135-150_ increased, there was a corresponding decrease in the association of TRAF6 with FHL2 (Figure 10*B*).

**Figure 11:**
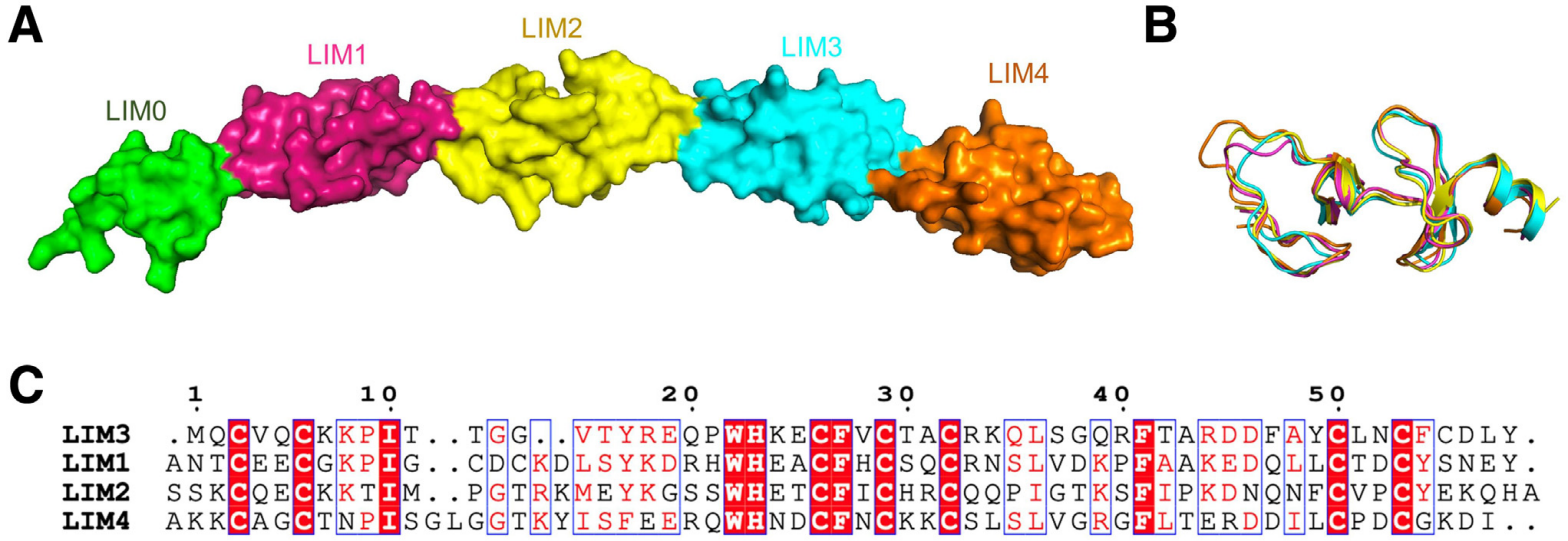
**The predicted structural characterization of FHL2.** (*A*) The full-length FHL2 protein consists of four and one-half LIM chains. (*B*) Four complete LIM domains have the same three-dimensional structure, with the RMSD ranging from 0.391 to 1.292. (*C*). The similarity of the 4 LIM domains is about 30%, and all of them contain conserved zinc finger motifs.

The full-length FHL2 protein comprises 4.5 LIM domains (Figure 11*A*). These 4 LIM domains exhibit an approximate 30% similarity among them and share conserved zinc finger motifs (Figure 11*B–C*). Importantly, all 4 complete LIM domains exhibit an identical 3-dimensional structure, with the root mean square deviation (RMSD) ranging from 0.391 to 1.292. To simulate the interaction between FHL2 and CASB_135-150_, we conducted molecular docking experiments involving different LIM domains of FHL2 and the CASB_135-150_ peptide. Among the resulting models, the binding of LIM4 and CASB_135-150_ exhibited the lowest HADDOCK score (Figure 10*C*). Notably, CASB_135-150_ likely interacts with LIM4 through hydrophobic interactions. At the interaction interface, amino acids L-P of CASB_135-150_ may directly engage in hydrogen bonds with F239 and F263 from LIM4 (Figure 10*C*). Notably, the amino acids LHLP of CASB_135-150_ is highly conserved among multiple mammals (Figure 2).

To further substantiate the role of CASB_135-150_ in FHL2/TRAF6-mediated NF-κB activation, a mutated version of CASB_135-150_, with the LHLP sequence altered to AAAA (CASB_135-150_-Mut), was generated. Subsequently, CASB_135-150_-Mut-HA was co-overexpressed with FHL2-Flag, and co-IP was carried out using the Flag antibody. As anticipated, the CASB_135-150_-mut failed to interact with FHL2 (Figure 10*D*). This was accompanied by an observed increase in the binding between TRAF6 and FHL2 compared with the interaction observed with CASB_135-150_ overexpression (Figure 10*D*). Additionally, it is noteworthy that this mutation effectively nullified the inhibitory effect of CASB_135-150_ on the production of inflammatory factors, as well as the translocation of p65 into the nucleus and NF-κB transcriptional activity in response to LPS (Figure 10*E–I*). Further, CASB_135-150_-Mut failed to reduce the NF-κB transcriptional activity induced by LPS (Figure 10*J*). Together, these observations suggest that CASB_135-150_ deconstructs FHL2/TRAF6 complex and inhibits inflammation response in macrophages.

### CASB_135-150_ Ameliorates NEC Intestinal Injury by Regulating NF-κB Signal

**Figure 12:**
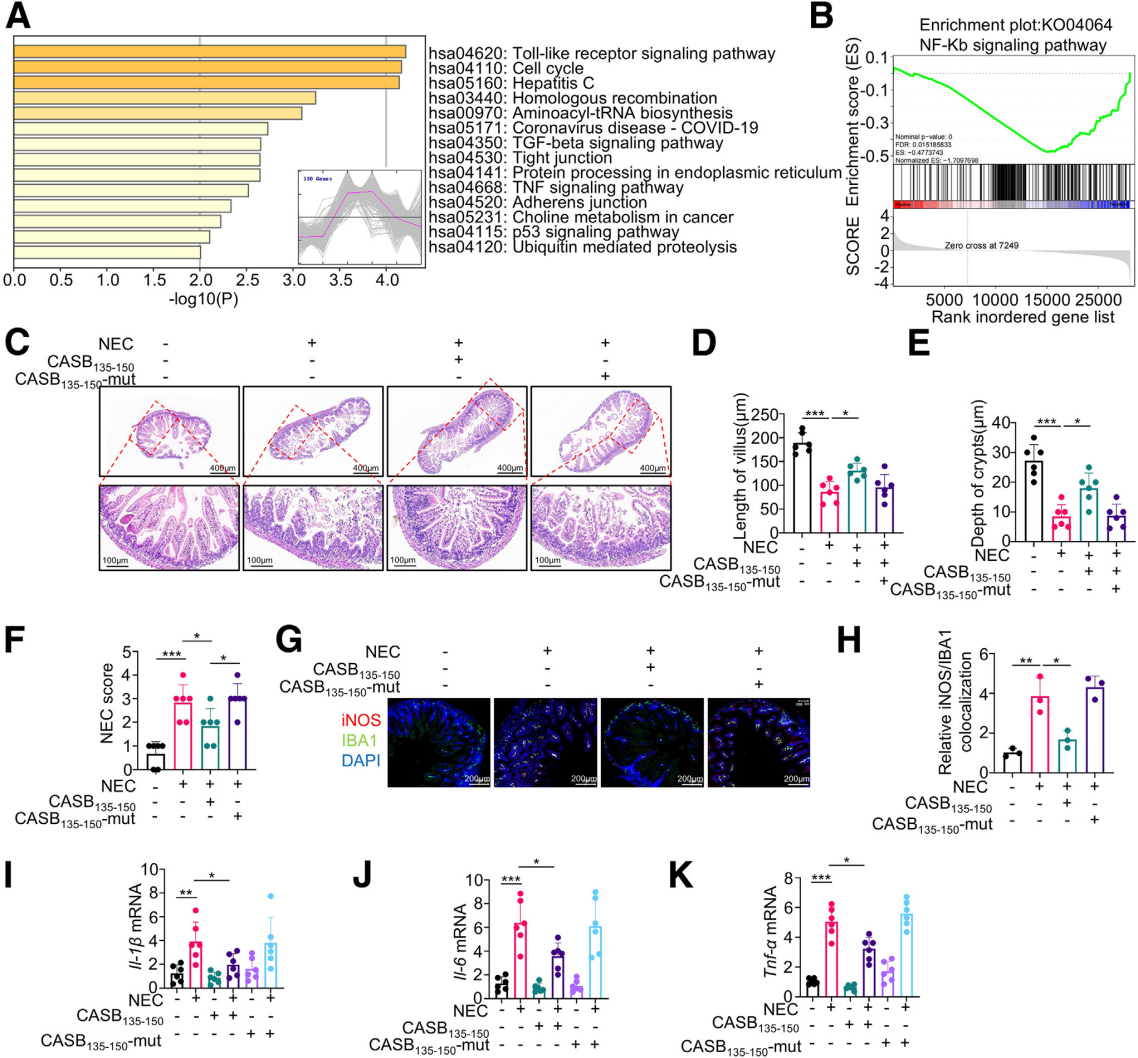
**CASB**_**135-150**_**ameliorates NEC intestinal injury by regulating NF-κB signal.** (*A*) The intestinal tissues from the control group, NEC group, and NEC+CASB_135-150_ group underwent transcriptomic analysis. A total of 530 genes within cluster 5 were analyzed for KEGG pathway analysis. (*B*) the GSEA between the NEC and NEC+CASB_135-150_ groups was conducted. (*C–F*) During the establishment of the NEC model, the neonatal rats were administered intraperitoneal injections of CASB_135-150_ or CASB_135-150_-Mut (20 mg/kg) on a daily basis. After 4 days, the ileums were harvested for H&E staining (*C*), villus length measurement (*D*), crypt depth assessment (*E*), and NEC scoring (*F*). n = 6. (*G–H*) Immunofluorescence analysis of iNOS and IBA1 in ileums was performed (*G*), and the quantification of iNOS/IBA1 colocalization was calculated (*H*). n = 3. (*I–K*) The expression of pro-inflammatory factors in ileums was also measured. n = 6. Data are presented as mean ± SD. Statistical significance was determined using 1-way ANOVA with Tukey’s post hoc test. ∗*P* < .05; ∗∗*P* < .01; ∗∗∗*P* < .001.

**Figure 13:**
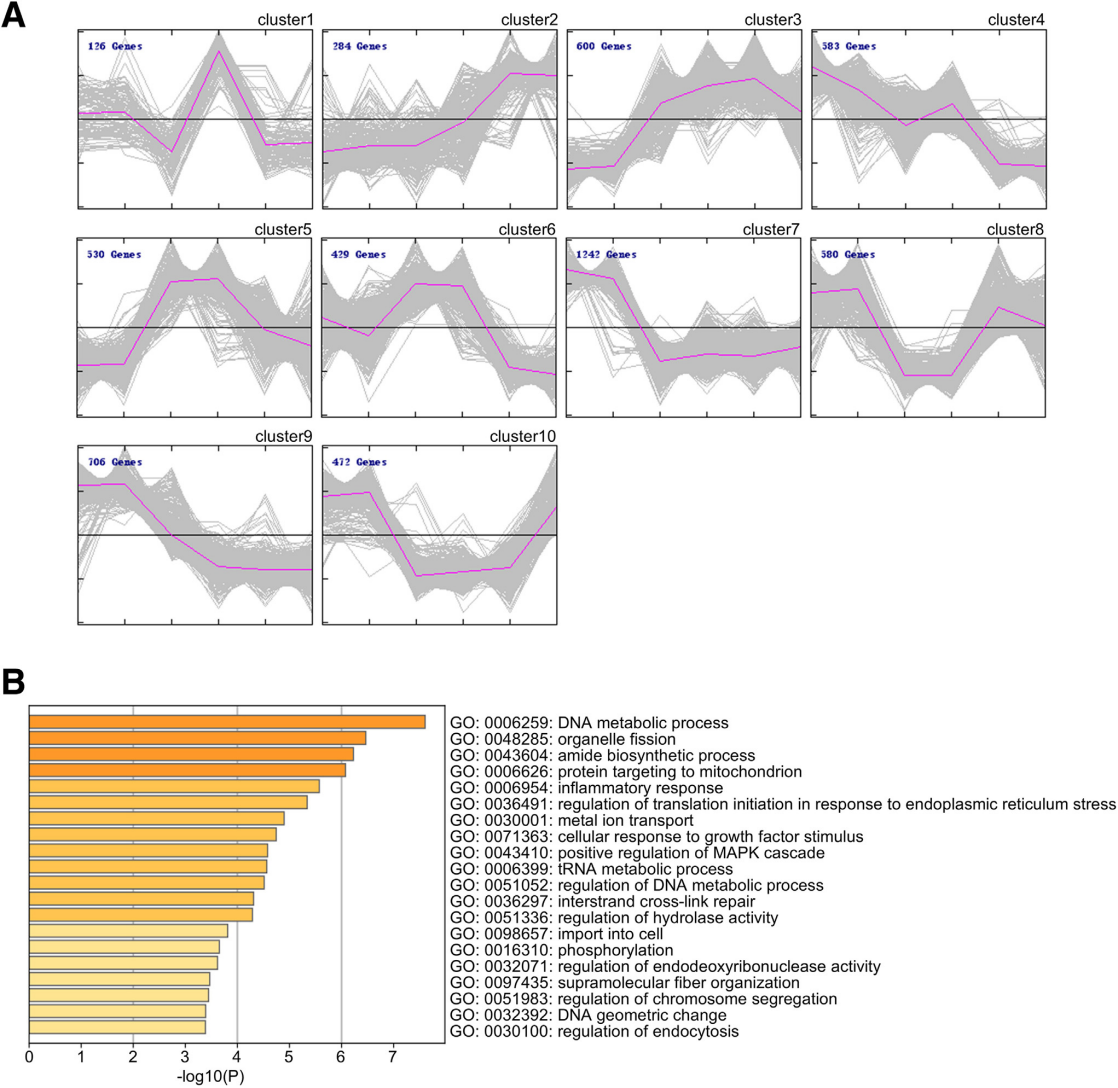
**The transcriptome alteration of small intestine upon CASB**_**135-150**_**treatment in vivo.** (*A*) The DEGs were divided into 10 distinct clusters through K-means clustering, based on their varying expression patterns. (*B*) A total of 530 genes within cluster 5 were analyzed for GO analysis.

To validate this mechanism *in vivo*, we administered CASB_135-150_ treatment to NEC rats and conducted RNA sequencing (RNA-seq) analysis on intestinal tissues, revealing a total of 5552 differentially expressed genes (DEGs) with a significance level of *P* < .05 (Figure 12*A*). To comprehensively characterize the dynamic changes in gene expression, we categorized these DEGs into 10 distinct clusters using K-means clustering (Figure 13*A*). Among these clusters, our focus was drawn to the group of genes displaying an upregulation in the NEC group and a subsequent downregulation in the NEC+CASB_135-150_ group (referred to as Clusters 5, encompassing a total of 530 genes). This pattern aligns with the expected characteristics of CASB_135-150_ functionality. Consequently, these genes were subjected to functional enrichment analysis. As anticipated, the Kyoto Encyclopedia of Genes and Genomes (KEGG) pathway analysis revealed that the Toll-like receptor signaling pathway ranked as the most significant pathway (Figure 12*A*). Moreover, the gene ontology (GO) analysis demonstrated a significant alteration in the process of inflammatory response (Figure 13*B*). Furthermore, through gene set enrichment analysis (GSEA) between the NEC and NEC+CASB_135-150_ groups, the results indicated that CASB_135-150_ treatment led to a negative regulation of the NF-κB signaling pathway (Figure 12*B*). Crucially, the in vivo study also demonstrated that the CASB_135-150_-Mut failed to ameliorate intestinal injury (Figure 12*C–F*) and macrophage inflammatory response in NEC rats (Figure 12*G–K*). Collectively, these findings suggest that CASB_135-150_ inhibits NF-κB-mediated inflammation by disrupting the FHL2/TRAF6 complex.

## Discussion

The precise impact and operational model of the human milk immune system on recipient infants continue to elude our understanding. Additionally, numerous unresolved remain regarding the role played by human milk peptides in this context. In the present study, we identified a milk-derived vesicle-borne peptide that swiftly suppresses pro-inflammatory responses in macrophages by disrupting the FHL2/TRAF6 complex, thereby inhibiting NF-κB signaling activity. Our discoveries unveil a novel mechanism through which human milk vesicle-encapsulated peptides contribute to innate immune homeostasis in infants.

Macrophages are highly plastic cells, and their phenotype can rapidly change in response to stimuli such as LPS.^26^ This underscores the necessity for a swift regulatory mechanism to orchestrate macrophage responses, particularly in preterm newborns with immature innate immunity, who are prone to exaggerated inflammatory responses. Macrophages are highly phagocytic immune cells that exhibit a strong tendency to engulf and internalize EVs. Evidence has shown that human milk EVs can be internalized by macrophages within 4 hours.^27^ Furthermore, bioactive components such as oligosaccharides and miRNA within milk EVs have the capacity to regulate macrophage inflammatory responses.^28^, ^29^, ^30^, ^31^ In our present study, CASB_135-150_ is the first peptide within human milk EVs identified to promptly regulate intestinal macrophage inflammation. Consequently, it is plausible that milk EVs serve as a highly effective conduit for maternal-infant immune communication, facilitating the transfer of bioactive components into macrophages to modulate innate immunity.

The full range of bioactivities exhibited by peptides in human milk typically becomes apparent only after they have been released from the parent proteins during digestion within the gastrointestinal tract.^17^^,^^32^ For instance, β-casein lacks immunomodulatory activity in its intact state but contains cryptic β-casomorphin peptides that, upon release through digestion, can induce an inflammatory immune response in the rat gut.^33^ These released peptides typically function through the proton-dependent peptide transporter, an intracellular vesicle-mediated transport system, or passive diffusion.^17^ Recently, a report indicated that a substantial portion of the bioactive peptides within human milk is conveyed via vesicles, potentially indicating a distinctive mode of action.^34^ Advances in proteomics have facilitated comprehensive investigations into the peptides contained within human milk EVs.^35^^,^^36^ However, the specific roles and mechanisms of these EV-carried peptides remain largely unknown. Our findings propose a novel model wherein milk-derived peptides potentially operate intracellularly via vesicular transport, significantly enhancing our comprehension of human milk peptides.

FHL2 is a member of a subgroup comprised of 5 homologous LIM domain-only proteins, known as FHLs.^37^ Initially considered to be primarily expressed in the heart, it has also been identified in striated muscle and a myeloid cell line.^37^, ^38^, ^39^ FHL2 has a dual role in the regulation of the NF-κB signaling pathway. Existing research has demonstrated that FHL2 exerts an inhibitory effect on NF-κB activity in the context of osteoclastogenesis.^40^ However, it plays a contrasting role as a positive regulator of NF-κB signaling in liver regeneration_25_ and promyogenesis.^41^ Notably, Dahan et al suggested that the depletion of FHL2 leads to a decrease in NF-κB activity induced by LPS in BMDMs, which aligns with our findings.^25^

FHL2 functions as an adaptor protein, with the ability to simultaneously bind various proteins, including cell surface receptors, cytosolic adaptor and structural proteins, kinases, and nuclear transcription factors.^23^^,^^42^ In HEK293 cells, FHL2 induces TRAF2-mediated activation of NF-κB by counteracting the suppressive effects of Tucan/Cardinal on NF-κB.^35^ In contrast, FHL2 inhibits RANK-mediated NF-κB activity in osteoclasts.^40^ Our data has shown that FHL2 enhance NF-κB signaling activity by binding and regulating TRAF6. Consequently, the multifaceted role of FHL2 can be attributed to its interaction with diverse proteins and its adaptability to function in various contexts. Interestingly, FHL2 overexpression also triggers a reduction of IL-6 production (Figure 9*A–B*). Given that FHL2 has a dynamic expression and dose-dependent interaction with certain proteins,^40^^,^^43^ this reduction in IL-6 production may be associated with the modulation of alternative pro-inflammatory signaling pathways. This phenomenon suggests that different levels of FHL2 may trigger diverse biological events, potentially resulting in consistent or contrasting outcomes.

The LIM domain within FHL2 represents a protein motif characterized by double zinc fingers, typically serving as an adaptor and scaffold for multimolecular complexes.^44^ Each TRAF protein contains a TRAF domain, and the TRAF domain of TRAF6 is sufficient for its recognition of FHL2.^40^ The interaction between FHL2 and TRAF6 significantly contributes to the stability of TRAF6.^25^ Our research has also indicated that following CASB_135-150_ treatment, there is a decrease in TRAF6 content (as shown in Figure 8*N*, *Q*). This effect may be attributed to FHL2’s influence on the posttranslational modifications of binding proteins, such as reducing (auto)ubiquitination.^42^ However, further evidence is required to elucidate the biochemical processes underlying the interaction between FHL2 and TRAF6.

The composition of human milk exhibits temporal variations and responds to the health statuses of both the mother and the infant.^45^ These variations confer multiple evolutionary advantages that significantly contribute to the survival and well-being of newborns. Our research findings substantiate this concept, revealing a higher abundance of CASB_135-150_ and β-casein-derived peptides in preterm milk compared with term milk. Additionally, CASB_135-150_ targets FHL2, an early intracellular signal transduction protein activated by TLR4. This targeting allows CASB_135-150_ to block inflammatory signals more efficiently upstream, limiting the subsequent amplification of the transcriptional cascade. Given the challenges mothers face in providing breast milk after preterm delivery, these peptides hold promise as potential components for the prevention of NEC.

Our study has several limitations. First, due to technical constraints, we have not fully investigated the impact of endogenous CASB_135-150_ depletion within EVs on NEC. Second, using intraperitoneal injection to assess peptide function may be less physiologically relevant compared with oral administration. However, peptides administered orally often degrade rapidly in the gastrointestinal tract, which limits the ability to evaluate their intraluminal effects. A more physiologically relevant approach, such as encapsulating CASB_135-150_ in a capsule with an intestine-specific polymer, is necessary for a comprehensive *in vivo* assessment of its function. Third, immunofluorescence showed that beta-casein fragments were also present in intestinal epithelial cells. Given the anti-inflammatory effects of CASB_135-150_ in epithelial cells observed in vitro, its potential role in NEC intervention within these cells should be considered. These issues will be addressed in future research.

In summary, our research identifies a specific peptide, CASB_135-150_, present in human milk EVs. This peptide interacts with FHL2, disrupting the FHL2/TRAF6 complex. This disruption leads to a rapid inhibition of NF-κB signaling activity and a subsequent reduction in inflammatory responses within intestinal macrophages. These findings provide valuable insights into the complex interactions between maternal feeding practices and the regulation of the infant’s innate immune system in the gastrointestinal tract.

## Materials and Methods

### Cell Culture

RAW264.7 and 293T cells were procured from the Chinese Academy of Sciences and cultured in Dulbecco’s modified Eagle medium (DMEM) (C3103-0500, VivaCell) supplemented with 10% fetal bovine serum (FBS) (10270, Gibco) and 1% penicillin/streptomycin (15140122, Thermo Fisher Scientific). IEC-6 cells were procured from the Chinese Academy of Sciences, and cultured in RPMI 1640 (VivaCell, C3001-0500) supplemented with 10% FBS, 10 μg/mL insulin (P3376-100IU, Beyotime), and 1% penicillin/streptomycin. All cells were incubated in a 5% CO_2_ environment at 37 °C. The cell lines were utilized for a maximum of 6 generations.

For the isolation and culture of BMDMs, we utilized C57BL/6 mice aged between 6 and 8 weeks. Femurs and tibias were harvested, and the bone marrow was flushed out with phosphate-buffered saline (PBS) (C3580-0500, VivaCell) using a 1 mL syringe. The collected cells were then passed through a 70 μm cell filter to eliminate cell clumps. Subsequently, 1 mL of Tris-NH4Cl solution was added, and the suspension was incubated for 10 minutes on ice to remove red blood cells. The isolated bone marrow cells were resuspended in DMEM supplemented with 10% FBS and 20 ng/mL macrophage colony-stimulating factor (M-CSF) (315-02, Peprotech). Finally, these cells were seeded into 6-well tissue culture plates.

### Peptide Synthesis and Treatment

The peptides CASB_135-150_ (DLENLHLPLPLLQPLM) and a scrambled peptide (DPPLHQLNLLEMLLLP) were conjugated at their N-termini to a carrier peptide derived from the HIV-TAT sequence (YGRKKRRQRRR) and synthesized by Shanghai Science Peptide Biological Technology Co, Ltd. For fluorescence tracking purposes, these peptides were attached at their N-termini to a FITC label. The neonatal rats received intraperitoneal injections of FITC-CASB_135-150_ (20 mg/kg); after 8 hours, the ileum tissue and ileum sections were subjected for fluorescence imaging.

In the experimental procedure, cells were pretreated with peptides at the specified concentrations for 1 hour, then the medium was replaced, the cells were washed with PBS, and fresh medium with LPS (L2630, Sigmaaldrich) was added. Subsequently, the cells were collected for real-time quantitative reverse-transcription polymerase chain reaction (qRT-PCR) and Western blot analysis.

### Cell Transfection and Lentiviral Packaging

Plasmids harboring 6×CASB_135-150_-HA, 6×CASB_135-150_ (Mut)-HA, or FHL2-3×Flag were generated using the pCDH vector. These plasmids were then subjected to lentivirus packaging. The HEK293T cells were plated into 10-cm dishes, and after reaching confluence, cells were transfected with 4 μg of the target plasmid (such as pCDH vector or pCDH-6×CASB_135-150_-HA), along with the packaging plasmids 3 μg psPAX2 and 1 μg pMD2.G using Lipofectamine 2000 (11668019, Invitrogen). Virus particles were collected 48 hours after transfection and then used for cell infection. To enhance the infection of each lentivirus, polybrene (5 μg/mL) was used for lentivirus infection. Following a 48-hour incubation period, the efficiency of expression was evaluated, and co-IP or qRT-PCR analyses were performed.

### qRT-PCR

Total RNA was extracted from cells using the EZ-press RNA Purification Kit (B0004D, EZBioscience). Total RNA was extracted from tissue using the EZ-press RNA Purification Kit (RN4, EZBioscience). Then, DNA was erased with DNase I, and cDNA was synthesized using HiScript III RT SuperMix kit (R323-01, Vazyme). Amplification of cDNA was measured with the ChamQ SYBR qPCR master mix (Q311-02/03, Vazyme) on the SLAN-96S PCR real-time PCR machine (Shanghai HONGSHI Medical Tech) according to the manufacturer’s instructions. Transcript levels were normalized to *Gapdh* levels. Primers used for qRT-PCR are shown in Table 3.

**Table 3:** The Primer Sequences Used for qPCR

RNA ID	Primer sequences
Mouse-*Gapdh*	F: 5'-CAACTCCCACTCTTCCACCT-3' R: 5'-GAGTTGGGATAGGGCCTCTC-3'
Mouse-*Il-6*	F: 5'-CTGGGAAATCGTGGAAATGAG-3' R: 5'-GACTCTGGCTTGTCTTTCTTGTTA-3'
Mouse-*Il-1β*	F: 5'-ACTCATTGTGGCTGTGGAGA-3' R: 5'-TTGTTCATCTCGGAGCCTGT-3'
Mouse-*Tnf-α*	F: 5'-GACCCCTTTACTCTGACCCC-3' R: 5'-AGGCTCCAGTGAATTCGGAA-3'
Rat-*Gapdh*	F: 5'-CCCCCAATGTATCCGTTGTG-3' R: 5'-TAGCCCAGGATGCCCTTTAGT-3'
Rat-*Il-6*	F: 5'-CATATGTTCTCAGGGAGATCTTGGA-3' F: 5'-CAGTGCATCATCGCTGTTCA-3'
Rat-*Il-1β*	F: 5'-CACCTTCTTTTCCTTCATCTTTG-3' F: 5'-GTCGTTGCTTGTCTCTCCTTGTA-3'
Rat-*Tnf-α*	F: 5'-CCCAGACCCTCACACTCAGAT-3' F: 5'-TTGTCCCTTGAAGAGAACCTG-3'

qPCR, Quantitative polymerase chain reaction.

### Human Milk Collection and EVs Isolation

Breast milk was collected from women who gave birth at Wuxi Children’s Hospital. In this study, preterm maternal colostrum refers to colostrum collected from mothers of newborns with a gestational age of 30 to 32 weeks, whereas term infant colostrum refers to colostrum collected from mothers of newborns with a gestational age of 39 to 40 weeks. Colostrum is defined as the breast milk produced 2 to 4 days after delivery. All human studies adhered to the Declaration of Helsinki principles. Informed consent was obtained from all participants. Participants were informed about the procedures and possible risks with the study.

The fresh human milk was collected for EVs isolation immediately, and informed consent was obtained from the participants at the time of milk sampling. The sample collection adhered to the legal requirements of the country in which the research was conducted. The obtained milk was subjected for EVs isolation using a Total Exosome Isolation Kit (4484453, Invitrogen) according to the manufacturer’s manual. The proteins derived from EVs were extracted using exosome protein lysate (UR33101, Umibio). The obtained protein underwent subsequent analysis through Western blot, NTA (ZetaView PMX110, Particle Metrix), and TEM (G2 Spirit FEI, Tecnai).

### Western Blot Analysis

After the specified treatments, total protein was extracted using RIPA lysate (P0013B, Beyotime Biotechnology) supplemented with a protease inhibitor cocktail (P1009, Beyotime Biotechnology) and a phosphatase inhibitor cocktail (P1046, Beyotime Biotechnology). The protein concentration was determined through a BCA assay (P0009, Beyotime Biotechnology). Standard protein electrophoresis was carried out, followed by the transfer of proteins to polyvinylidene fluoride (PVDF) membranes (IPVH00010, Millipore). After a 1-hour blocking step, the PVDF membranes were used for protein analysis. Images were captured using a ChemiDoc System (Tanon 5200 Multi).

The primary antibodies employed were as follows: Rabbit anti-Phospho-NF-κB p65 (3033S, Cell Signaling Technology, 1:1000 dilution), Rabbit anti-NF-κB p65 (8242S, Cell Signaling Technology, 1:1000 dilution), Rabbit anti-IκBα (4812S, Cell Signaling Technology, 1:1000 dilution), Rabbit anti-Phospho-IκBα (2859S, Cell Signaling Technology, 1:1000 dilution), Rabbit anti-β-Actin (4970T, Cell Signaling Technology, 1:1000 dilution), Rabbit anti-FLAG tag (14793S, Cell Signaling Technology, 1:1000 dilution), Rabbit anti-HA tag (51064-2-AP, Proteintech, 1:1000 dilution), Rabbit anti-FHL2 (ab202584, Abcam, 1:1000 dilution), Rabbit anti-TRAF6 (67591S, Cell Signaling Technology, 1:1000 dilution), Rabbit anti-β-casein (ab205301, Abcam, 1:1000 dilution), Rabbit anti-TSG101 (28283-1-AP, Proteintech, 1:1000 dilution), Rabbit anti-GM130 (11308-1-AP, Proteintech, 1:1000 dilution), and Rabbit anti-CD9 (20597-1-AP, Proteintech, 1:1000 dilution).

### Co-IP Assay

Cells were transfected with the designated plasmids for 48 hours, following which the total protein was collected using an IP lysis buffer (containing Tris pH 7.5, 150 mM NaCl, and 0.5% NP-40 (85124, Thermofisher). Co-IP was conducted using either anti-FLAG or anti-HA M2 Magnetic Beads (A36797, 88836, Thermo Fisher), following the manufacturer’s instructions. After 6 washes with PBS, the samples were eluted using 2 × protein loading buffer (P0288, Beyotime) and separated by SDS-PAGE.

### Extraction of Lamina Propria Mononuclear Cells From Rat Ileum

The ileum was removed from rats and washed with PBS (02-020-1A, VivaCell) to remove fat. The intestine was then longitudinally opened, washed in Hank’s balanced salt solution (HBSS) (14175095, Gibco) containing 10% FBS (10270, Gibco), and cut into 0.5-cm segments. The tissue was placed in 10 mL of HBSS with 10% FBS and vigorously shaken to remove the supernatant. Next, 10 mL of calcium-free and magnesium-free HBSS (14175095, Gibco) containing 2 mM EDTA (60-00-4, Sigma) was added, and the tubes were placed in a 37 °C water bath, shaken vigorously for 15 minutes, and the supernatant was discarded. This step was repeated with 10 mL of calcium- and magnesium-free HBSS (14175095, Gibco), followed by vigorous shaking and discarding the supernatant. The remaining tissue was then placed in complete RPMI 1640 (C3001-0500, VivaCell) containing 1.25 mg/mL collagenase D (11088858001, Roche), 0.85 mg/mL collagenase V (C9263, Sigma), 1 mg/mL dispase (17105041, Thermo), and 30 U/mL DNase (04536282001, Roche). Digestion occurred in a shaking water bath for 30 to 40 minutes until the tissue was fully digested. During incubation, tubes were vigorously shaken every 5 to 10 minutes, and the final supernatant was filtered through a 70 μm mesh (352350, Corning).

### Flow Cytometry

The harvested BMDMs were suspended in a FACS buffer (2% FCS in PBS). Subsequently, the cells were treated with an Anti-Fc receptor antibody from BD Biosciences (553141) at 4 °C for 15 minutes. Following this, the cells were stained with APC anti-mouse CD86 Antibody (105012, Biolegend, 1:200 dilution), and PE anti-mouse CD206 (MMR) Antibody (141706, Biolegend, 1:100 dilution). The samples were analyzed using a BD FACSCanto II from BD Biosciences, and the data were analyzed using FlowJo software.

The harvested lamina propria mononuclear cells (LPMCs) were suspended in a FACS buffer (2% FCS in PBS). The cells were then treated with an Anti-Fc receptor antibody (550271, BD Biosciences) at 4 °C for 15 minutes. Following this, the cells were stained with the following antibodies: FITC anti-CD45 Antibody (202205, BioLegend, 1:200 dilution), APC anti-F4/80 Antibody (123115, BioLegend, 1:200 dilution) and PerCP/Cy5.5 anti-CD11b/c Antibody (201819, BioLegend, 1:200 dilution). We then centrifuged and discarded the supernatant, added 100 μL of fixation and permeabilization solution (554714, BD), resuspended the cells, incubated at 4 °C in the dark for 40 minutes, centrifuged and discarded the supernatant, added 150 μL of permeabilization wash buffer (554714, BD), centrifuged and discarded the supernatant, then added the diluted Alexa Fluor 700 anti-TNF-α antibody (NBP1-19532AF700, Novus Biologicals, 1:100 dilution), resuspended the cells, and incubated in the dark for 60 minutes. Then we centrifuged and discarded the supernatant, then added 300 μL of PBS to resuspend the cells. The samples were analyzed using a BD FACSCanto II (BD Biosciences), collecting a total of 50,000 events per sample. FlowJo 10.8.1 software was used for analysis. The gating strategy is depicted in Figure 3*B*. Initially, lymphocytes were gated using FSC-A/SSC-A and FSC-A/FSC-H to exclude cell debris and aggregates. CD45+ immune cells were gated based on SSC-A/CD45. Subsequently, cells positive for F4/80 and CD11b/c were defined as macrophages, and finally, M1 type macrophages were gated based on TNF-α expression.

### Immunofluorescence

For the immunofluorescence experiments, cells were cultured on glass coverslips, fixed with 4% paraformaldehyde at room temperature for 30 minutes, and then blocked with 5% bovine serum albumin (BSA) (ST023, Beyotime Biotechnology) for 1 hour at room temperature. They were subsequently incubated with Rabbit anti-iNOS (13120S, CST), Rabbit anti-CD86 (13395-1-AP, Proteintech), and Rabbit anti-NF-κB p65 (8242S, CST) at a 1:500 dilution overnight at 4 °C. After washing with PBS, the cells were incubated with Alexa Fluor 488 or 568 conjugated secondary antibodies (A-11008, A-11012, Thermo Fisher) at a 1:500 dilution for 1 hour while avoiding exposure to light. Cell nuclei were stained with 4′,6-diamidino-2-phenylindole (DAPI, P0131, Beyotime Biotechnology).

For immunofluorescence staining of tissue sections, we placed the washed sections onto a plastic rack, immersed in boiling antigen retrieval solution, maintained boiling for 15 minutes, then kept it warm for 15 minutes and allowed it to cool naturally. After washing with PBS, the sections were deparaffinized and blocked with 5% BSA for 1 hour at room temperature. Subsequently, they were incubated with Rabbit anti-iNOS (ab283655, Abcam) at a 1:500 dilution overnight at 4 °C. After washing with PBS, we added 100 μl of Goat anti-rabbit IgG H&L (HRP) (ab205718, Abcam, 1:2000) and incubated at 37 °C for 30 minutes. After washing with PBS, we added 100 μl of 488-tyramide conversion reagent (Bry-try488, runnerbio) to each section, and incubated at room temperature for 10 to 30 minutes. We then repeated the heat antigen retrieval and blocking steps. After washing with PBS, they were incubated with Rabbit anti-IBA1 (ab178847, Abcam) at a 1:500 dilution overnight at 4 °C. After washing with PBS, we added 100 μl of cy5-tyramide conversion reagent (Bry-trycy5, runnerbio) to each section. The following steps were consistent with the immunofluorescence staining of cells. The results of the immunofluorescence staining were visualized using an Olympus BX53 microscope (BX53, Olympus).

### Dual Luciferase Reporter Gene Experiment

The luciferase reporter construct pGMNF-κB-Lu from Genomeditech was co-transfected with the Renilla luciferase construct into RAW264.7 cells using the lipofectamine 3000 reagent (L3000075, Thermo Fisher) following the manufacturer's instructions. Following the designated treatment, the Firefly & Renilla Assay Kit (abs60341, Absin) was employed for the dual luciferase reporter assay. The treated cells were harvested as per the kit’s instructions and subsequently analyzed using the Fluoroskan Ascent FL fluorescence and chemiluminescence analyzer (Thermo). The Firefly luciferase signals were normalized using the Renilla luciferase signals. The data is presented as the ratio of Firefly to Renilla luciferase activities, with the control group set as the reference point at one.

### Animal Study

The SD rats utilized in this study were obtained from SPF Biotechnology Co., Ltd. Newborn pups (1 day old) were nourished with Abbott puppy formula every 3 hours through an orogastric feeding catheter. To induce NEC, these neonates were subjected to stress by exposure to hypoxia (5% O2 and 95% N2) and a 4°C for 10 minutes after each of their three daily feedings. For hypertonic feeding, neonatal rats were gavaged with a formula containing 15 g Similac 60/40 (Ross Pediatrics) in 75 mL Esbilac (Pet-Ag), providing 836.8 kJ/kg per day. Feedings started at 0.1 mL and were gradually increased to a maximum of 0.4 mL per feeding. For peptide intervention, CASB_135-150_ or scrambled peptide (20 mg/kg/day) was administered during the gavage. Healthy control groups consisted of naturally born neonatal rats nursed by their mothers. At the conclusion of the experiment on day 5, the animals were sacrificed, and their intestines were collected for later analysis.

Histological assessments of hematoxylin and eosin (H&E)-stained intestinal sections were performed by a pathologist to evaluate ileum damage using a previously published NEC scoring system.^46^ The system assessed the degree of intestinal injury on a scale from “0 to 4,” as follows: 0: No histological damage; 1 (Mild): Slight submucosal and/or lamina propria separation; 2 (Moderate): Moderate separation of the submucosa and/or lamina propria and/or edema in the submucosa and muscular layers; 3 (Severe): Severe separation of the submucosa and/or lamina propria and/or severe edema in the submucosa and muscular layers with regional villous sloughing; 4 (Necrosis): Loss of villi and necrosis. Scores ≥2 are defined as NEC.

For immunofluorescence of β-casein, neonatal rats were fasted for 4 hours and then gavaged with saline (200 μL) containing human milk EVs (5 × 10^9^). After 2 hours, the ileums were harvested for IBA1 and β-casein immunofluorescence. For peptide tracing *in vivo*, neonatal rats were intraperitoneally injected with FITC-labeled CASB_135-150_ (20 mg/kg). After 8 hours, the ileums were harvested for frozen sectioning and FITC fluorescence imaging.

The random number table method was employed for the grouping of rats, and the double-blind method was used for evaluation. Only the supervisor was aware of the group allocation at the different stages of the experiment. No exclusions of experimental units or data points were made in the analysis for each experimental group. The minimum number of animals was determined through power analysis and following the principles of the 3Rs. Rats were anesthetized using isoflurane and euthanized by CO_2_ asphyxiation. All animal experiments adhered to the Guide for the Care and Use of Laboratory Animals published by the National Institutes of Health (NIH Publications No. 85-23, revised 1996) and were approved by the Medical Ethics Committee of the Ethics of Animal Experiments at Jiangnan University.

### scRNA-seq Analysis

The scRNA-seq data of the human neonatal small intestine affected by NEC was obtained from the Zenodo database (https://doi.org/10.5281/zenodo.5813397). The 12 scRNA-seq datasets were combined using the Seurat package in R. After filtering with thresholds of nCount_RNA >1900, percent.mt <30, and nFeature_RNA >1000, batch correction was performed using the Harmony package. We then applied a resolution of 0.2 and used UMAP for dimensionality reduction. Marker genes for each cluster were identified using FindAllMarkers, and cell types were annotated based on these marker genes using the SingleR package in R.

### RNA-seq

To elucidate the alterations in gene expression within the intestines following NEC and CASB_135-150_ treatment, ileums were extracted from 3 groups: the control group (neonatal rats nursed by their mothers), the NEC group (NEC model with scrambled peptide), and the NEC+CASB_135-150_ group (NEC model with CASB_135-150_ peptide). Total RNA was extracted from the mouse small intestine using TRIzol. High-throughput RNA sequencing was conducted by Genedenovo Biotechnology Co, Ltd on the HiSeqTM 4000 sequencing platform (Illumina). Differential expression from RNA-seq experiments was determined by fragments per kilobase of transcript per million mapped reads (FPKM), normalizing for gene length and sequencing depth. Genes with a *P*-value < .05 in the comparison were deemed significant DEGs.

### Statistical Analysis

Statistical analysis and data visualization were carried out using GraphPad Prism 8.0 (GraphPad Inc). Statistical analyses were conducted using the Student’s *t*-test, as well as the 1-way or 2-way analysis of variance (ANOVA) test, as appropriate. The data in this study were presented as the mean ± standard deviation (SD). Statistical significance was defined as *P* < .05. The number of experimental replicates or animals used is indicated in the corresponding legend.
